# Natural Merosesquiterpenes Activate the DNA Damage Response via DNA Strand Break Formation and Trigger Apoptotic Cell Death in p53-Wild-Type and Mutant Colorectal Cancer

**DOI:** 10.3390/cancers13133282

**Published:** 2021-06-30

**Authors:** Apisada Jiso, Philipp Demuth, Madeleine Bachowsky, Manuel Haas, Nina Seiwert, Daniel Heylmann, Birgit Rasenberger, Markus Christmann, Lea Dietrich, Thomas Brunner, Riyanti, Till F. Schäberle, Anuchit Plubrukarn, Jörg Fahrer

**Affiliations:** 1 Division of Food Chemistry and Toxicology, Department of Chemistry, Technical University of Kaiserslautern, 67663 Kaiserslautern, Germany; jiso.apisada@gmail.com (A.J.); pdemuth@rhrk.uni-kl.de (P.D.); madele.bach@online.de (M.B.); manhaas@rhrk.uni-kl.de (M.H.); seiwert@rhrk.uni-kl.de (N.S.); 2 Department of Pharmacognosy and Pharmaceutical Botany, Faculty of Pharmaceutical Sciences, Prince of Songkla University, Hat-Yai, Songkhla 90112, Thailand; anuchit.pl@psu.ac.th; 3 Rudolf Buchheim Institute of Pharmacology, Justus Liebig University Giessen, 35392 Giessen, Germany; Daniel.Heylmann@pharma.med.uni-giessen.de; 4 Institute of Toxicology, University Medical Center Mainz, 55131 Mainz, Germany; rasebi00@uni-mainz.de (B.R.); mchristm@uni-mainz.de (M.C.); 5 Biochemical Pharmacology, Department of Biology, University of Konstanz, 78464 Konstanz, Germany; lea.dietrich@uni-konstanz.de (L.D.); thomas.brunner@uni-konstanz.de (T.B.); 6 Institute for Insect Biotechnology, Justus-Liebig-University Giessen, 35392 Giessen, Germany; Riyanti@bio.uni-giessen.de (R.); Till.F.Schaeberle@agrar.uni-giessen.de (T.F.S.); 7 Faculty of Fisheries and Marine Science, Jenderal Soedirman University, Purwokerto 53122, Indonesia; 8 Fraunhofer Institute for Molecular Biology and Applied Ecology (IME), Branch for Bioresources, 35392 Giessen, Germany

**Keywords:** colorectal cancer, chemotherapy, tumor suppressor p53, apoptosis, natural compounds, DNA damage

## Abstract

**Simple Summary:**

Bowel cancer is a serious disease, which affects many people worldwide. Unfortunately, the disease is often diagnosed in an advanced stage, which impairs the chance of survival. Furthermore, resistance to therapy occurs frequently. Thus, novel therapeutic approaches are required to improve cancer therapy. Here, we studied whether merosesquiterpenes might be useful for cancer treatment. These compounds occur in marine sponges and were isolated by our group. We were able to identify three compounds with potent cytotoxic activity in different cell lines established from human large bowel cancer. Our experiments provided evidence that the compounds cause DNA damage and trigger cell death, so-called mitochondrial apoptosis, which was attested in cancer cells with expression of wild-type and mutated p53 tumor suppressor. Finally, we show that merosesquiterpenes also kill intestinal tumor organoids, an ex vivo model of large bowel cancer.

**Abstract:**

Colorectal cancer (CRC) is a frequently occurring malignant disease with still low survival rates, highlighting the need for novel therapeutics. Merosesquiterpenes are secondary metabolites from marine sponges, which might be useful as antitumor agents. To address this issue, we made use of a compound library comprising 11 isolated merosesquiterpenes. The most cytotoxic compounds were smenospongine > ilimaquinone ≈ dactylospontriol, as shown in different human CRC cell lines. Alkaline Comet assays and γH2AX immunofluorescence microscopy demonstrated DNA strand break formation in CRC cells. Western blot analysis revealed an activation of the DNA damage response with CHK1 phosphorylation, stabilization of p53 and p21, which occurred both in CRC cells with p53 knockout and in p53-mutated CRC cells. This resulted in cell cycle arrest followed by a strong increase in the subG1 population, indicative of apoptosis, and typical morphological alterations. In consistency, cell death measurements showed apoptosis following exposure to merosesquiterpenes. Gene expression studies and analysis of caspase cleavage revealed mitochondrial apoptosis via *BAX*, *BIM*, and caspase-9 as the main cell death pathway. Interestingly, the compounds were equally effective in p53-wild-type and p53-mutant CRC cells. Finally, the cytotoxic activity of the merosesquiterpenes was corroborated in intestinal tumor organoids, emphasizing their potential for CRC chemotherapy.

## 1. Introduction

Marine sponges are an important source of a plethora of bioactive natural compounds [1]. Merosesquiterpenes belong to these secondary metabolites produced by marine sponges, which were reported to display various biological activities including anti-inflammatory and antibacterial properties [1]. Ilimaquinone (IQ) is a well-studied member of this class. There is increasing evidence that IQ also exerts antiproliferative and/or cytotoxic effects as attested by its growth inhibition of prostate, liver, lung, and pancreatic cancer cells [2]. More recently, IQ was reported to induce apoptosis in oral squamous cell carcinoma, which was attenuated by transient knockdown of the tumor suppressor p53 [3]. In another study, IQ was shown to promote TRAIL-induced cell death in colorectal cancer (CRC) cells via upregulation of TRAIL death receptor expression, which was associated with moderate induction of reactive oxygen species (ROS) [4]. Increased mitochondrial ROS levels were also observed in lung cancer cells exposed to IQ, leading to apoptosis induction [5]. Moreover, it was reported that IQ causes the accumulation of p53 in two CRC cell lines and can induce cell death on its own [6]. Very recently, IQ has been identified as an activator of the DNA damage response (DDR) within a large screen consisting of 296 natural compounds [7]. Consistent with this finding, IQ was toxic in pancreatic cancer cells without anticancer drug treatment [7]. Smenospongine (SP) is a structurally related compound, which was hardly studied with regard to its putative cytotoxic effects in cancer cells until now. It was shown that SP inhibits the growth of different leukemia cell lines and increases the subG1 population, indicative of apoptosis [8]. A more recent study provided evidence that SP eliminates breast cancer stem cells by activating p38 and AMPK-signaling pathways [9]. Collectively, these studies suggest that merosesquiterpenes may have the potential to kill cancer cells and could offer a novel therapeutic option in the treatment of CRC.

CRC is a frequently occurring malignant disease, which is causally linked to various genetic, lifestyle, and dietary risk factors [10,11,12]. CRC incidence and mortality are increasing in younger people under 50 years of age, particularly in Europe and the United States [13,14]. The treatment of CRC involves surgical resection, chemotherapy, radiation, and, in metastatic disease, targeted therapies [15]. Despite recent progress in CRC therapy, patients with the advanced and metastatic disease still face low 5-year survival rates, highlighting the need for novel therapeutics. These promising therapeutic strategies include inhibitors of tumor cell metabolism such as α-lipoic acid and others [16,17], natural or synthetic small molecules targeting oncogenic signaling mediated by mTOR, AKT, or STAT3 [18], and inhibitors of the DDR [19].

To address this issue, we made use of a compound library comprising 11 naturally occurring merosesquiterpenes. First, the identity and purity of all isolated compounds were characterized by NMR spectroscopy. A cytotoxicity screening was conducted in three CRC cell lines with wild-type p53 (HCT116, RKO) or mutated p53 (HT29), allowing for the calculation of IC_50_ values. As a next step, the formation of DNA damage and ROS production was evaluated by the alkaline Comet assay, immunofluorescence microscopy, and flow cytometry using the most promising compounds. Activation of the DDR and effects on cell cycle progression were assessed by Western blot analysis and flow cytometry. Furthermore, cell death induction and apoptosis triggered by selected merosesquiterpenes were studied by flow cytometry, Western blot detection, quantitative PCR, and microscopy. The role of p53 was addressed by using isogenic p53 knockout cells and p53 knockdown by siRNA. Finally, the results were translated into a murine tumor organoid model using viability assays, phase-contrast, and fluorescence microscopy.

## 2. Materials and Methods

### 2.1. Compounds

**Figure 1:**
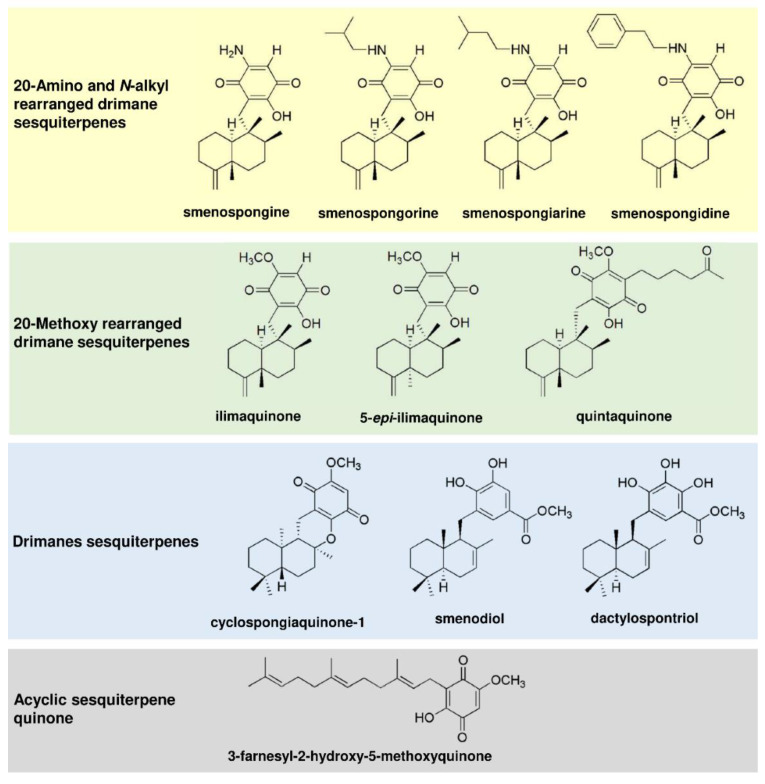
Chemical structures of merosesquiterpenes used in this study.

The merosesquiterpenes used in this study (Figure 1) were isolated from the sponges *Haliclona sp.* (smenospongine) [20] and *Verongula rigida* (smenospongorine, smenospongiarine, smenospongidine, ilimaquinone, 5-epi-ilimaquinone, quintaquinone, cyclospongiaquinone-1, smenodiol, dactylospontriol, and 3-farnesyl-2-hydroxy-5-methoxy quinone) [21], as described. All tested compounds were directly used with no additional purification steps. The structures were reauthenticated using spectroscopic analysis, particularly NMR spectroscopy. The spectral data were identical to those reported previously [20,21,22,23,24,25,26,27,28,29]. The NMR spectrum of each tested compound revealed almost no signals of impurity. The compounds were stored at −20 °C until experiments were carried out. Please see Supporting Information for the isolation and ^1^H NMR spectral data (Figures S1–S11). The anticancer drug 5-fluorouracil (5-FU) was from Medac (Wedel, Germany) and provided by the pharmacy of the University Medical Center Mainz.

### 2.2. Cell Culture and Treatments

The human CRC cell line HCT116 and isogenic p53-deficient HCT116 cells were provided by Dr. Bert Vogelstein (John Hopkins University, Baltimore, MD, USA). HT29 and RKO cells were provided by the Institute of Toxicology, University Medical Center Mainz, Germany. HCT116 and RKO cells were maintained in DMEM and HT29 cells in RPMI1640 medium supplemented with 10% fetal calf serum and 1% penicillin/streptomycin at 37° C in a humidified atmosphere of 5% CO_2_. Media and supplements were obtained from Gibco Life Technologies (Darmstadt, Germany) and PanBiotech (Aidenbach, Germany). All cell lines were mycoplasma negative, as confirmed by routine PCR testing. The merosesquiterpenes were dissolved in DMSO as a 10 mM stock solution and added to the culture medium reaching a final concentration from 0–100 µM. DMSO served as solvent control (0 µM). Cells were exposed to the compounds for up to 72 h, as indicated.

### 2.3. Transient Transfection with siRNA

Knockdown of p53 was performed using siGENOME SMARTpool siRNA bought from Dharmacon (Lafayette, CO, USA). Non-sense, scrambled RNA, also purchased at Dharmacon, was used as control. Transfections were carried out, as reported previously [30]. Briefly, HT29 cells were transfected with 20 nM siRNA using Lipofectamine™ RNAiMAX (Thermo Fisher Scientific, Darmstadt, Germany) for 24 h before exposure to ilimaquinone for 48 h. Successful knockdown of p53 was verified by Western blot analysis.

### 2.4. Preparation of Cell Lysates

Whole-cell extracts were generated as described [31]. After treatment for 48 h, cells were harvested, and whole-cell lysis was performed using a buffer containing 25 mM Tris-HCl pH 8.0, 5 mM EDTA, 1 mM DTT, 0.5 M NaCl supplemented with cOmplete™ protease inhibitor cocktail (Roche Diagnostics, Mannheim, Germany). After incubation for 15 min at 4 °C on a rotating platform, extracts were clarified by centrifugation (10 min, 10,000× *g*) and protein content was determined using Bradford assay.

Blue extracts were prepared as published elsewhere [32]. Briefly, cells were treated with the compounds for 24 h, as indicated and harvested in 1 × Lämmli loading buffer.

### 2.5. SDS–PAGE and Immunoblot Analysis

Western blot analysis was essentially performed as described [33]. Equal protein amounts were separated by SDS–PAGE, followed by wet blot transfer onto a nitrocellulose membrane (PerkinElmer, Rodgau, Germany). Membranes were blocked with 5% nonfat dry milk in TBS/0.1% Tween-20 (TBS-T) for 1 h at RT. Subsequently, primary antibody incubation was carried out overnight at 4° C, followed by three washing steps in TBS/0.1% Tween-20. Membranes were then incubated with appropriate secondary antibodies for at least 1 h at RT. After three washing steps in TBS-T, proteins were detected with Western Lightning^®^ Plus-ECL (PerkinElmer, Rodgau, Germany) using a c300 chemiluminescence imager (azure biosystems, Dublin, CA, USA).

### 2.6. Antibodies

The following primary antibodies were used: Hsp90α/β (F8, mouse monoclonal; Santa Cruz Biotechnology, Dallas, TX, USA, no. sc-13119), p53 (DO-1, mouse monoclonal; Santa Cruz, no. sc-126), p21 (C-19, rabbit polyclonal; Santa Cruz, no. sc-397), γH2AX (Ser139, rabbit monoclonal; Abcam, Cambridge, United Kingdom, no. ab81299), cleaved caspase-3 (rabbit monoclonal, Cell Signaling Technology, Danvers, MA, USA, no. 9661) and cleaved caspase-9 (rabbit monoclonal, Cell Signaling Technology, no. 7237), phospho-CHK1 (Ser345; rabbit monoclonal; Cell Signaling Technology, no. 2348), Bim (rabbit monoclonal, Cell Signaling Technology, no. 2933). Secondary antibodies conjugated with horseradish–peroxidase were purchased from Santa Cruz (anti-mouse, no. K2818) and Cell Signaling Technology (anti-rabbit, no. 7074).

### 2.7. Isolation of RNA, cDNA Synthesis, and Quantitative Real-Time PCR (qPCR)

Gene expression analysis was performed as described [34]. Total RNA was isolated using the NucleoSpin^®^ RNA Kit (Macherey-Nagel, Düren, Germany), and concentrations were determined using a NanoDrop^TM^ 2000 spectrophotometer (Thermo Scientific, Waltham, MA, USA). 0.5 µg of total RNA was transcribed into cDNA using the Verso cDNA Synthesis Kit (Thermo Scientific, Dreieich, Germany). qPCR was performed with the SensiMix^TM^SYBR Green and Fluorescein Kit (Bioline, London, UK) and the CFX96^TM^ Real-Time PCR Detection System (Biorad, München, Germany), with the primers detailed below (Table 1). In all three experiments, qPCR was conducted using biological and technical triplicates. The analysis was performed using CFX Manager^TM^ Software (BioRad, Hercules, CA, USA). Non-transcribed controls were included in each run. Finally, the expression of genes of interest was normalized to *GAPDH* and *ACTB*. The solvent control was set to one.

### 2.8. Measurement of ROS Formation

HCT116 cells grown in 6-well plates were exposed to smenospongine and ilimaquinone at the desired concentration for 24 h. As positive control, cells were treated with 200 µM H_2_O_2_ in PBS for 20 min. ROS generation was essentially determined as reported [35]. Cells were washed twice with PBS and loaded with CM-H_2_DCFDA (Life Technologies, Darmstadt, Germany) in phenol red- and serum-free medium for 30 min. The cells were washed with PBS, detached by Trypsin/EDTA treatment, and collected by centrifugation. Finally, the cell pellets were resuspended in PBS, analyzed using a FACSCanto II flow cytometer (BD Biosciences, Heidelberg, Germany), and evaluated using FACSDiva software 6.0 (BD Biosciences).

### 2.9. Alkaline Comet Assay

HCT116 cells grown in 6-well plates were exposed to smenospongine and ilimaquinone for 24 h, as indicated. Etoposide (10 µM) served as positive control. Formation of DNA strand breaks and AP sites was assessed using the alkaline Comet assay as reported [36], with a cell lysis step (50 min), followed by alkaline DNA unwinding step (20 min) prior to electrophoresis under alkaline conditions. Following DNA staining with propidium iodide, samples were analyzed by using an Axioskop 2 fluorescence microscope (Zeiss, Jena, Germany). In total, 50 cells per slide were scored using the Comet Assay IV software (Perceptive Instruments, Bury St Edmunds, UK).

### 2.10. Cell Cycle Analysis

Cell cycle distribution was studied as described previously [37]. Briefly, CRC cells were exposed to the compounds as indicated for 24 h and 48 h, respectively. Attached and detached cells were collected. Cell pellets were washed with ice-cold PBS, followed by a precipitation step with ethanol overnight at −20 °C. Subsequently, cell pellets were resuspended in PBS containing RNase A (20 µg/mL; Sigma, Deisenhofen, Germany) and incubated for one hour. Finally, propidium iodide (PI; Sigma) was added in a final concentration of 10 µg/mL, and cells were analyzed using a FACSCanto II flow cytometer (BD Biosciences, Heidelberg, Germany). Cell cycle distribution was assessed with FACSDiva software 6.0 (BD Biosciences).

### 2.11. Cell Death Analysis

Cell death induction was studied by AnnexinV-FITC and PI staining as reported [38]. In short, CRC cells were exposed to the compounds for 48 h, as indicated. Attached and detached cells were harvested following Trypsin/EDTA digestion by centrifugation, washed in PBS, and resuspended in binding buffer (10 mM HEPES pH 7.4, 140 mM NaCl, 2.5 mM CaCl_2_, 0.1% BSA) containing AnnexinV-FITC (Miltenyi Biotec, Bergisch Gladbach, Germany). After incubation on ice for 15 min, a binding buffer containing PI (50 µg/mL) was added, and samples were subject to flow cytometry using a FACSCanto II (BD Biosciences). Gating of living cells (Annexin V/PI double negative), early apoptotic cells (Annexin V-positive, PI-negative), and late apoptotic/necrotic cells (Annexin V/PI-double positive) and data evaluation was performed with FACSDiva software 6.0 (BD Biosciences).

### 2.12. Cell Viability Assay and Determination of IC_50_ Values

To analyze the effects of the compounds on cell viability, CRC cells were grown in white 96-well plates overnight and incubated with increasing concentrations for 72 h, as indicated. Cell viability was then assessed with Cell Titer-Glo^®^Luminescent Cell Viability Assay (Promega, Mannheim, Germany) according to the manufacturer’s instructions using a Fluoroskan™ FL 96-well plate reader (Thermo Fisher, Dreieich, Germany). IC_50_-values were determined by GraphPad Prism 7.0 software as reported previously [39]. To this end, concentrations were transformed into the log scale, plotted against the cell viability and the curve was fitted by nonlinear regression with variable slope, providing the IC_50_ values.

### 2.13. Analysis of Cell Morphology

CRC cells were treated with the compounds as indicated for up to 48 h. Cell morphology was monitored with a Leica DMi1 microscope equipped with an MC170 camera (Leica, Wetzlar, Germany). Images were acquired with LAS EZ software 3.4.0 (Leica).

### 2.14. Cultivation of Murine Tumor Organoids and Assessment of Viability

Murine tumor organoids were grown in 3D culture using Cultrex^®^ basement membrane extract (BME) (R&D Systems, Inc., Minneapolis, MN, USA) and maintained as described previously [40]. Tumor organoids were collected by removing the culture medium and adding 500 µL of cold DMEM. The BME was destroyed with a pipette tip and tumor organoids were collected in a 15 mL tube. After centrifugation at 100 g for 5 min and removal of the supernatant, 5 mL TrypLE was added, and tumor organoids were digested at 37 °C for 10 min. TrypLE was inactivated by adding 10 mL of DMEM and cells were again centrifuged at 100 g for 5 min. Cells were resuspended in 1 mL basal organoid culture medium, consisting of advanced DMEM/F12, 10 mM HEPES, 100 U/mL penicillin, 100 mg/mL streptomycin, 20 mg/mL nystatin, 1 mM N-acetyl cysteine (all from Sigma, Schnelldorf, Germany), 0.1% BSA (Carl Roth, Karlsruhe, Germany), 2 mM L-glutamine, 1× B27 supplement, 1× N2 supplement (all from Life Technologies, Darmstadt, Germany). Using a 96 well plate, 2.4 × 10^3^ cells were seeded per well by applying 8 µL droplets of a 1:4 mixture of basal culture medium and BME. After polymerization for 30 min, 80 µL of complete culture medium were added, consisting of basal organoid culture medium supplemented with 50 ng/mL murine EGF, 100 ng/mL murine Noggin (all from Peprotech, Hamburg, Germany), 10 mM Nicotinamide, 500 nM A83-01 (all from Sigma, Schnelldorf, Germany), 10 μM Y-27632 (MedChemExpress, Monmouth Junction, NJ, USA) and mR-Spondin-1 conditioned medium in a final concentration of 20% (*v*/*v*). Subsequently, tumor organoids were cultivated at 37 °C and 5% CO_2_ for 72 h. After exchanging the culture medium, tumor organoids were exposed to smenospongine and ilimaquinone in the desired concentrations as well as 5-FU as the positive control for another 72 h.

Tumor organoid viability was determined by the MTS assay as described [30]. After discarding the medium, 80 µL basal organoid culture medium containing 75 µg/mL MTT were added and tumor organoids were incubated for 1 h at 37 °C. BME was solubilized by adding 20 µL of 10% SDS solution to each well and incubating for 30 min at 37 °C. Absorbance was measured using a microplate reader at 490 nm. Background absorbance was estimated by measuring tumor organoids without added MTT and subtracted from all values. Solvent treated organoids were defined as 100% viable.

### 2.15. Immunofluorescence Microscopy of CRC Cells and Tumor Organoids

HCT116 cells grown on coverslips were treated with smenospongine and ilimaquinone as indicated and incubated for 24 h. γH2AX foci formation was analyzed by immunofluorescence microscopy as described elsewhere [32]. After fixation with paraformaldehyde and methanol, cells were blocked in PBS containing 5% BSA and 0.3% Triton X-100 for 1 h. Subsequently, the samples were incubated with a primary antibody directed against γH2AX (diluted 1:1000; see Section 2.6.) for 1 h. After several washing steps, incubation with a secondary antibody conjugated with Alexa Fluor 488 (1:400; Life Technologies, Darmstadt, Germany) was performed for 1 h. Finally, the samples were mounted using Vectashield^®^ containing DAPI (VectorLabs, Burlingame, CA, USA). Analysis was carried out with a Zeiss Axio Observer 7 microscope equipped with an Axiocam 305 mono (Carl Zeiss Microscopy, Jena, Germany). Images were acquired and processed with ZEN software (Carl Zeiss Microscopy, Jena, Germany).

Tumor organoids were seeded in 96-well plates and cultivated, as mentioned above. Tumor organoids were then treated with smenospongine and ilimaquinone in the desired concentrations as well as 5-FU as the positive control for 72 h. Microscopic determination of cell death in tumor organoids was performed by PI/ Hoechst 33342 co-staining, as described elsewhere [41]. Briefly, the organoid culture medium was removed after treatment and PBS containing PI and Hoechst 33342 at a final concentration of 10 μg/mL each was added for 30 min. The staining solution was exchanged for basal organoid culture medium before analysis by fluorescence microscopy, as described in the last paragraph.

### 2.16. Statistics

Experiments were performed independently three times, except otherwise stated. Representative experiments are displayed. Values are presented as means + standard error of the means (SEM) using GraphPad Prism 7.0 software. Statistical analysis was performed using a two-sided Student’s *t*-test and statistical significance was defined as *p* < 0.05.

## 3. Results

### 3.1. Cytotoxicity Screening of Merosesquiterpenes in Human CRC Cell Lines

**Figure 2:**
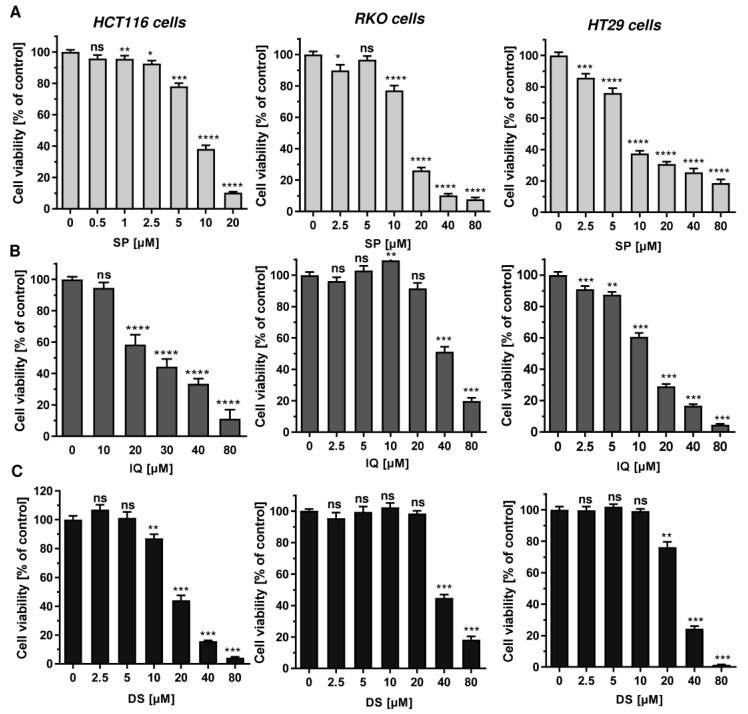
Cytotoxicity of selected merosesquiterpenes in CRC cell lines: (**A**–**C**) CRC cells (HCT116, RKO, HT29) were treated with increasing concentration of SP (panel A), IQ (panel B) and DS (panel C) for 72 h. Viability was determined using a luminescent ATP test. Data are presented as mean + SEM (*n* = 3). Not significant (ns): *p* > 0.05; * *p* < 0.05; ** *p* < 0.01; *** *p* < 0.001; **** *p* < 0.0001.

First, 11 merosesquiterpenes (Figure 1) were analyzed with regard to their cytotoxic potential in 3 colorectal cancer (CRC) cell models, displaying either microsatellite instability (HCT116, RKO) or chromosomal instability (HT29) [42]. Smenospongin (SP) caused a concentration-dependent reduction in cell viability, which was most pronounced in HCT116 cells, followed by HT29 and RKO cells (Figure 2A). Ilimaquinone (IQ) was somewhat less potent than SP and displayed the highest cytotoxicity in HT29 cells (Figure 2B). Dactylospontriol (DS) was most effective in HCT116 cells and exerted similar cytotoxicity in RKO and HT29 cells (Figure 2C). The calculated IC_50_ values ranged from 8 µM to 44 µM for these three compounds in all CRC cell lines (Table 2). The other compounds tested revealed either weaker cytotoxicity throughout the CRC cell lines (e.g., smenospongorine and smenodiol) or only showed moderate efficacy in HCT116 cells and were therefore excluded from the analysis in RKO and HT29 cells. Two compounds (smenospongiarine and 3-farnesyl-2-hydroxy-5-methoxyquinone) showed a lack of cytotoxicity in HCT116 cells and were therefore not considered further (Table 2). Taken together, SP, IQ, and DS were identified as the most promising merosesquiterpenes in our initial cytotoxicity screening.

### 3.2. Activation of the DNA Damage Response and Impact on Cell Cycle Progression

**Figure 3:**
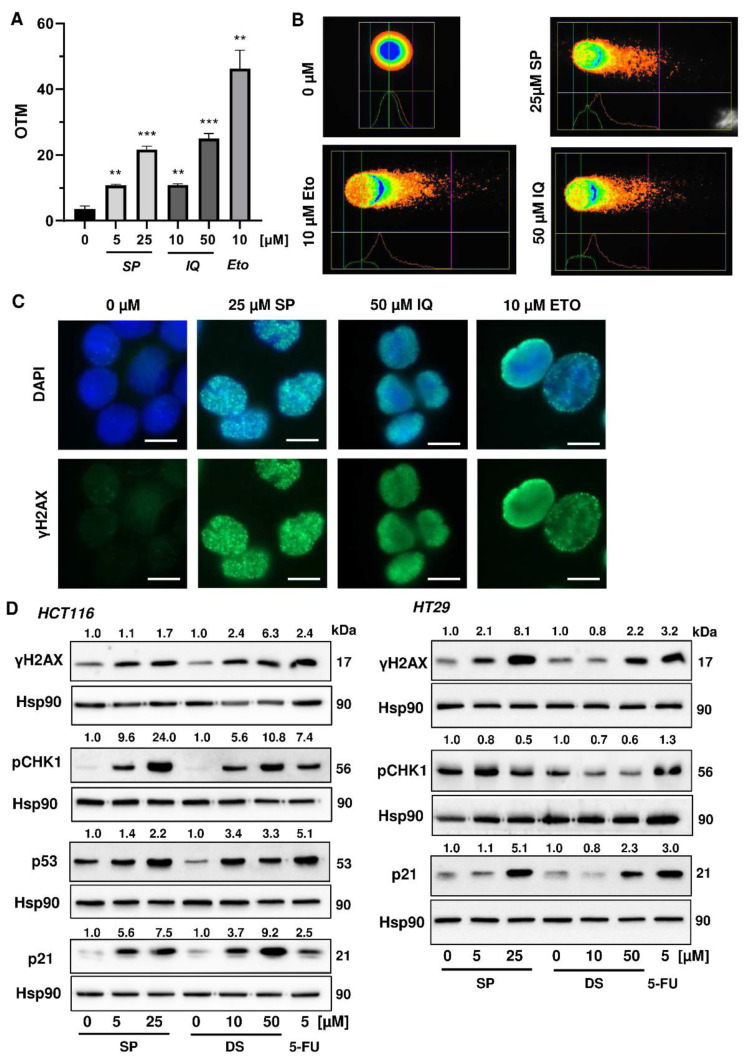
Induction of DNA strand brakes and DNA damage response in CRC cells after treatment with merosesquiterpenes: (**A**) HCT116 cells were treated with SP or IQ as indicated for 24 h. The anticancer drug etoposide was included as positive control. DNA strand break induction was determined by the alkaline Comet assay. Data are presented as mean + SEM (*n* = 3). ** *p* < 0.01, *** *p* < 0.001; (**B**) representative images of Comet assay shown in A; (**C**) HCT116 cells were treated as described in (**A**). Formation of the DNA damage marker γH2AX was visualized by immunofluorescence microscopy. Nuclei are depicted in blue, while γH2AX is shown in green. Representative pictures are displayed. Scale bar: 10 µm; (**D**) HCT116 cells were treated with SP or IQ, as indicated, for 24 h. The anticancer drug 5-FU was included as positive control. Samples were analyzed by SDS–PAGE and Western Blot detection of γH2AX, p-CHK1, p53, and p21. Hsp90 served as loading control. Representative blots are shown together with the densitometry evaluation.

**Figure A1:**
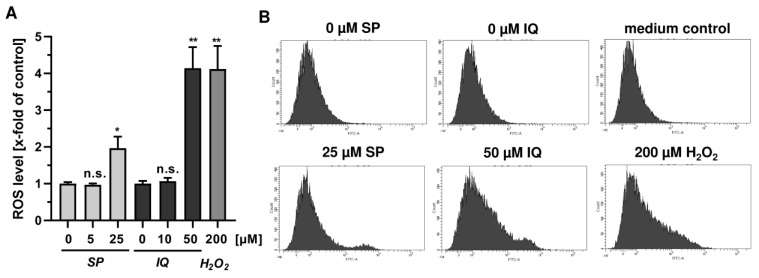
Effects of merosesquiterpenes on cellular ROS production and DNA damage response in CRC cells after 24 h: (**A**) flow-cytometry-based measurements of ROS formation in HCT116 cells challenged with increasing concentrations of SP and IQ, respectively. H_2_O_2_ was included as positive control. Data are given as mean + SEM (*n* = 4). Not significant (ns): *p* > 0.05; * *p* < 0.05; ** *p* < 0.01; (**B**) representative histograms of ROS analysis shown in (**A**).

Next, we studied whether the merosesquiterpenes cause DNA damage and trigger the DNA damage response (DDR). To this end, we used HCT116 cells with wild-type p53 protein and HT29 cells that express p53 with a hot spot mutation in the DNA binding domain [43]. First, DNA strand break formation was analyzed using the alkaline Comet assay, which detects DNA strand breaks and alkali labile sites. A concentration-dependent increase in DNA damage levels was observed after treatment with both SP and IQ (Figure 3A,B). These findings were substantiated by immunofluorescence microscopy of phosphorylated histone 2AX (γH2AX), a well-established marker for DNA strand breaks that was induced by both merosesquiterpenes (Figure 3C). Western blot analysis revealed a concentration-dependent increase of γH2AX after SP and DS exposure in HCT116 cells after 24 h (Figure 3D). Furthermore, phosphorylation of the checkpoint kinase 1 (CHK1) was detected (Figure 3D and Figure A1C), also reflecting DDR activation. Consistent with this finding, a simultaneous accumulation of p53 and its downstream target p21 was observed. Interestingly, these effects were even more pronounced than that of the positive control 5-FU, an antimetabolite used for CRC chemotherapy. Similar findings were obtained in p53-mutated HT29 cells, except for CHK1 phosphorylation that was rather unaffected after 24 h (Figure 3B). Furthermore, ROS formation was measured in CRC cells after 24 h, revealing increased ROS levels following treatment with high concentrations of SP and IQ (Figure A1A,B).

**Figure 4:**
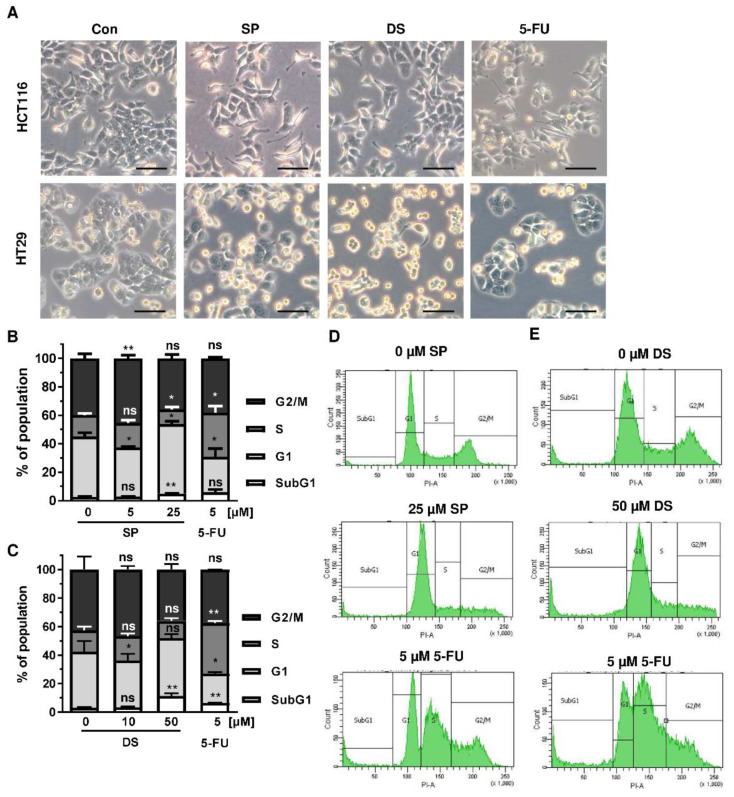
Effects on morphology and cell cycle distribution in CRC cells after treatment with merosesquiterpenes: (**A**) HCT116 and HT29 cells were incubated with solvent control, 25 µM SP, 50 µM DS and 5 µM 5-FU for 24 h. Cell morphology was observed by light microscopy. Representative pictures are shown. Scale bar: 25 µm; (**B**,**C**) cell cycle analysis in HCT116 cells challenged with increasing concentrations of SP (**B**) and DS (**C**)**,** respectively, for 24 h, and 5-FU served as positive control. Data are given as mean + SEM (*n* ≥ 3). Not significant (ns): *p* > 0.05; * *p* < 0.05; ** *p* < 0.01; (**D**,**E**) representative histograms of cell cycle analysis shown in (**B**,**C**).

**Figure A2:**
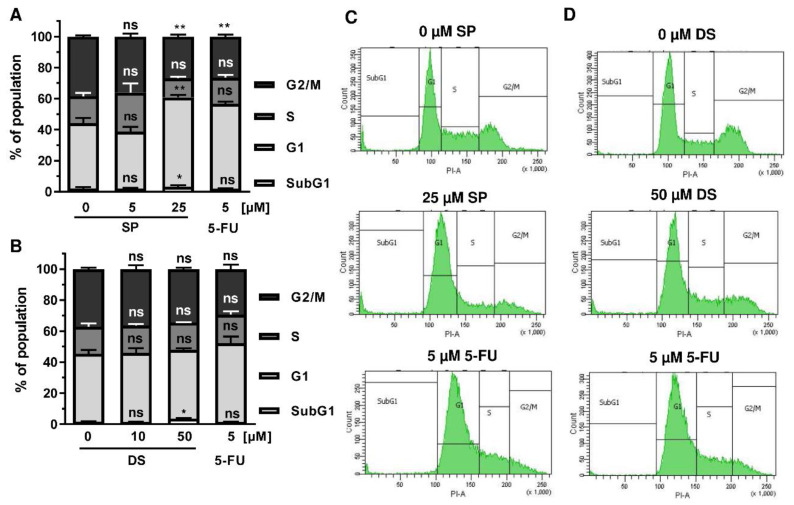
Effect of merosesquiterpenes on cell cycle distribution in HT29 cells after 24 h: (**A**,**B**) cell cycle analysis in HT29 cells challenged with increasing concentrations of SP (**A**) and DS (**B**), respectively, for 24 h, and 5-FU served as positive control. Data are given as mean + SEM (*n* = 4). Not significant (ns): *p* > 0.05; * *p* < 0.05; ** *p* < 0.01; (**C**,**D**) representative histograms of cell cycle analysis shown in (**A**,**B**).

Since p53 controls cell cycle progression via p21, the cell cycle distribution was analyzed in both CRC cell lines following treatment with merosesquiterpenes for 24 h. In both CRC cell lines, SP and DS reduced cell density and/or promoted cell rounding, as shown by microscopy (Figure 4A). In HCT116 cells, SP caused an accumulation of cells in the G2-M phase at low concentration, while a high concentration resulted in decreased S-phase population and higher G1 population (Figure 4B,D). DS displayed similar effects at low concentration but increased subG1 at high concentration, the latter being indicative of apoptotic cell death (Figure 4C,E). In HT29 cells, SP at high concentration caused a G1 arrest with a decreased number of cells in the S-phase (Figure A2A,C), whereas DS moderately induced subG1 population (Figure A2B,D).

**Figure 5:**
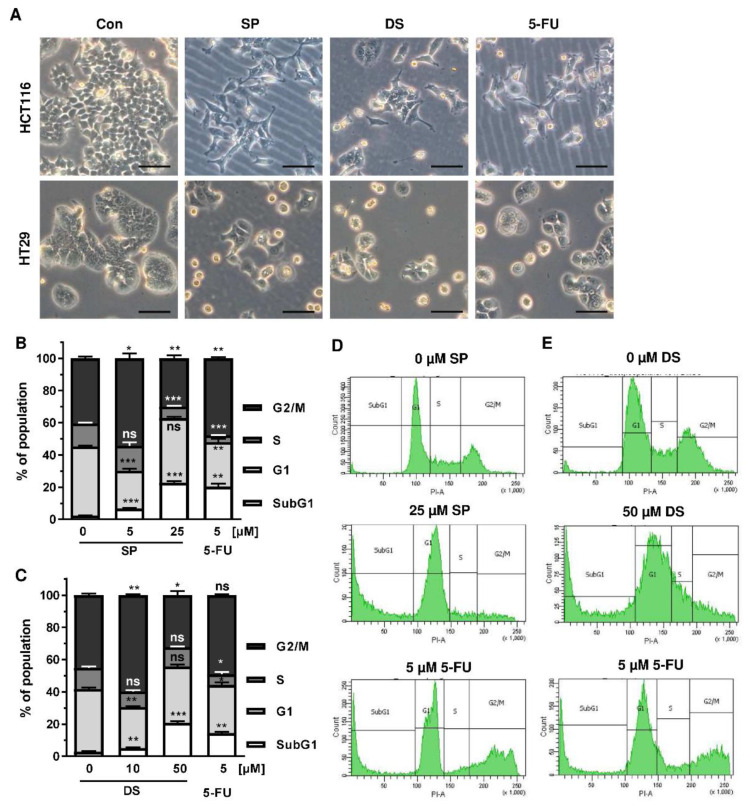
Morphological changes and cell death induction by merosesquiterpenes in CRC cells: (**A**) HCT116 and HT29 cells were incubated with solvent control, 25 µM SP, 50 µM DS and 5 µM 5-FU for 48 h. Cell morphology was observed by light microscopy. Representative pictures are shown. Scale bar: 25 µm; (**B**,**C**) cell cycle analysis in HCT116 cells treated with increasing concentrations of SP (**B**) and DS (**C**), respectively, for 48 h, and 5-FU served as positive control. Data are given as mean + SEM (*n* ≥ 3). Not significant (ns): *p* > 0.05; * *p* < 0.05; ** *p* < 0.01; *** *p* < 0.001; (**D**,**E**) representative histograms of cell cycle analysis performed in (**B**,**C**).

**Figure A3:**
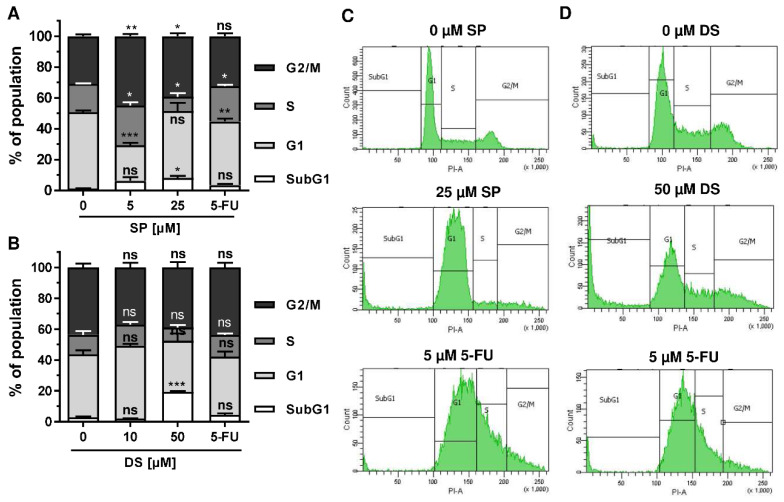
Effect of merosesquiterpenes on cell cycle distribution in HT29 cells after 48 h: (**A**,**B**) cell cycle analysis in HT29 cells treated with increasing concentrations of SP (**A**) and DS (**B**), respectively, for 48 h, and 5-FU served as positive control. Data are given as mean + SEM (*n* = 4). Not significant (ns): *p* > 0.05; * *p* < 0.05; ** *p* < 0.01; *** *p* < 0.001; (**C**,**D**) representative histograms of cell cycle analysis performed in (**A**,**B**).

We then analyzed how prolonged exposure to SP and DS over 48 h affects morphology and cell cycle distribution in HCT116 and HT29 cells. Microscopy revealed drastic morphological changes with increased cell rounding and detachment of cells, strongly suggesting cell death induction (Figure 5A). In agreement with these observations, SP and DS caused a huge increase of the subG1 population in HCT116 cells at high concentrations (Figure 5B–E), which was also detected in HT29 cells (Figure A3A–D). Treatment with lower concentrations of both compounds induced a pronounced G2-M arrest in the tested CRC cell lines. Interestingly, the positive control 5-FU exerted also potent cytotoxic effects in HCT116 cells (Figure 5), whereas 5-FU had little impact on HT29 cells, as attested by lack of subG1 (Figure A3).

In conclusion, our results demonstrated that merosesquiterpenes cause DNA strand breaks, activate the CHK1-p53-p21 mediated DDR, affect cell cycle progression in p53-mutant and p53-wild-type cells, and increase the subG1 population indicative of cell death.

### 3.3. Cell Death Induction by Merosesquiterpenequinones and Impact of p53

**Figure 6:**
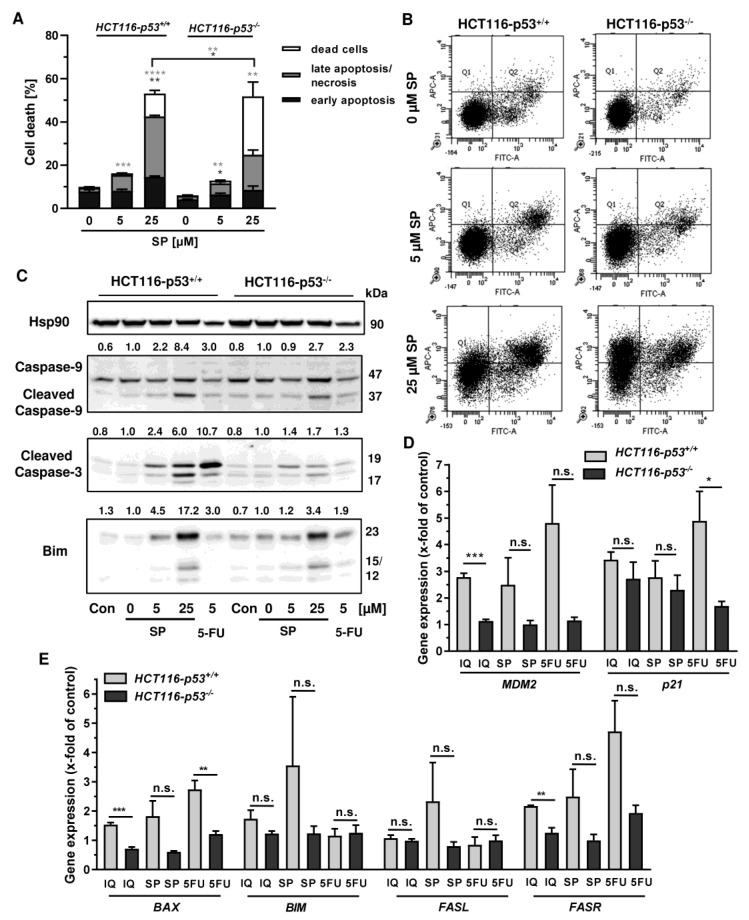
Impact of p53 status on merosesquiterpenes triggered cell death in CRC cells: (**A**) isogenic HCT116-p53^+/+^ and HCT116-p53^−/−^ cells were exposed to increasing concentrations of SP for 48 h. Cell death induction was assessed by Annexin V-FITC/PI staining and flow cytometry. Data are shown as mean + SEM (*n* = 3). Not significant (ns): *p* > 0.05; * *p* < 0.05; ** *p* < 0.01; *** *p* < 0.001; **** *p* < 0.0001; (**B**) representative dot plots of (**A**); (**C**) isogenic HCT116 cell lines differing in their p53 status were exposed to increasing concentration of SP for 48 h. 5-FU was included as positive control and untreated cells as the corresponding negative control. Samples were subject to SDS–PAGE, followed by Western blot analysis of cleaved caspase-3, cleaved caspase-9, and Bim. Hsp90 was included as the loading control. Representative blots are shown including the densitometric evaluation; (**D**) expression levels of *MDM2* and *p21* in p53-proficient and -deficient HCT116 cells treated with 50 µM IQ, 25 µM SP and 5 µM 5-FU for 24 h. Gene expression was assessed by q PCR (*n* = 3); (**E**) expression levels of proapoptotic genes (*BAX, BIM, FASL, FASR*) in HCT116 cells differing in their p53-status treated as described in (**D**). Gene expression was assessed by qPCR (*n* = 3). Not significant (ns): *p* > 0.05; * *p* < 0.05; ** *p* < 0.01; *** *p* < 0.001.

**Figure A4:**
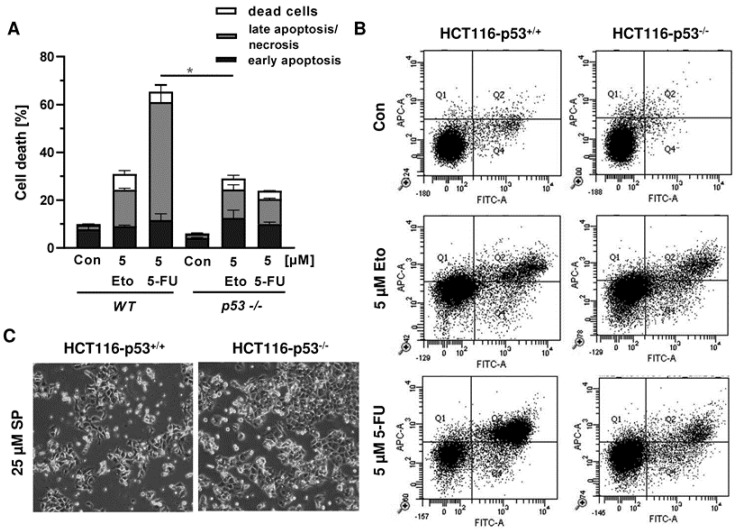
Impact of p53 status on anticancer drug triggered cell death in CRC cells: (**A**) isogenic HCT116-p53^+/+^ and HCT116-p53^−/−^ cells were exposed to 5 µM 5-FU and 5 µM Etoposide (Eto) for 48 h. Cell death induction was assessed by Annexin V-FITC/PI staining and flow cytometry. Data are shown as mean + SEM (*n* = 3). Not significant (ns): *p* > 0.05; * *p* < 0.05; ** *p* < 0.01; (**B**) representative dot plots of measurements performed in (**A**). (**C**) Morphological changes in HCT116-p53^+/+^ and HCT116-p53^−/−^ cells challenged with 25 µM SP for 48 h.

In order to investigate the cell death mechanism and the role of p53, we made use of isogenic HCT116 cells proficient and deficient for p53 (HCT116-p53^+/+^ vs. HCT116-p53^−/−^). Both cell lines were exposed to increasing concentrations of smenospongine for 48 h, and cell death was analyzed by Annexin V/PI staining. SP induced both early apoptosis and late apoptosis/necrosis, which was significantly reduced in HCT116 p53 k.o. cells (Figure 6A,B). However, a marked increase in dead cells (i.e., only PI positive) was observed in HCT116-p53^−/−^ cells after treatment with SP at high concentration. In addition, 5-FU triggered cell death in a p53-dependant manner in HCT116 cells as expected, whereas the p53 status did not affect cell killing by etoposide (Figure A4A,B). Microscopic inspection revealed a similar ratio of round and detached cells in both isogenic HCT116 cell lines but a somewhat higher cell density in p53 k.o. cells (Figure A4C).

**Figure A5:**
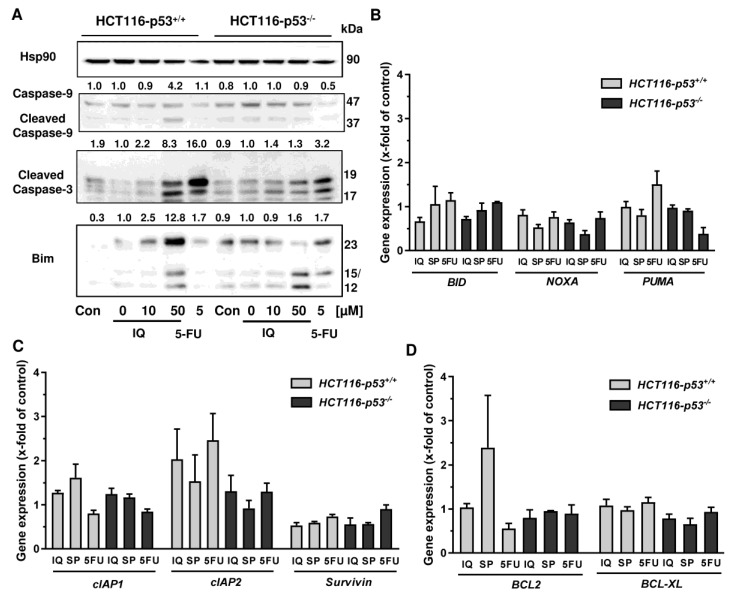
Impact of p53 status on caspase cleavage, proapoptotic and antiapoptotic gene expression in CRC cells upon merosesquiterpene treatment: (**A**) isogenic HCT116 cell lines differing in their p53 status were exposed to increasing concentration of IQ for 48 h, and 5-FU was included as positive control and untreated cells as the corresponding negative control. Samples were subject to SDS–PAGE, followed by Western blot analysis of cleaved caspase-3, cleaved caspase-9, and Bim. Hsp90 was used as loading control. Representative blots are shown including densitometric evaluation; (**B**) expression levels of the proapoptotic genes *BID, NOXA*, and *PUMA* in p53-proficient and -deficient HCT116 cells treated with 50 µM IQ, 25 µM SP and 5 µM 5-FU for 24 h. Gene expression was assessed by qPCR (*n* = 3); (**C**) expression levels of the antiapoptotic genes *cIAP1*, *cIAP2*, and *Survivin* determined, as described in A; (**D**) expression levels of the antiapoptotic genes *BCL2* and *BCL-XL* determined as described in A.

Since both Annexin/PI staining and analysis of cell cycle distribution indicated apoptosis induction by SP, the cleavage of the caspase-3 and caspase-9 was assessed by Western blot detection. The experiments demonstrated a concentration-dependent cleavage of both caspases upon SP treatment, which was strongly attenuated in p53-deficient HCT116 cells, particularly for caspase-9 (Figure 6C). Similar to this finding, caspase-9 cleavage by 5-FU treatment was completely blocked in HCT116 p53 k.o. cells (Figure 6C). IQ also caused caspase-9 and caspase-3 cleavage, which was much more prominent in HCT116 cells with p53 expression (Figure A5A). Furthermore, the proapoptotic BH3-only protein Bim was analyzed, revealing a concentration-dependent increase in HCT116 p53 WT cells upon both SP and IQ treatment (Figure 6C and Figure A5A). This effect was strongly attenuated in HCT116 p53 k.o. cells. Interestingly, the p53 status had no impact on Bim levels after challenge with 5-FU, which were found moderately elevated in both isogenic HCT116 cell lines (Figure 6C and Figure A5A).

The impact of p53 on the mode of cell death triggered by merosesquiterpenes was further studied by gene expression analysis using qPCR. The p53 negative feedback regulator *MDM2* was induced in HCT116-p53^+/+^ cells by both SP and IQ, as well as the positive control 5-FU, which was completely abolished in p53 k.o. cells (Figure 6D). Furthermore, the cell cycle regulator *p21* was substantially upregulated by all test compounds. Interestingly, this effect was strongly repressed in HCT116-p53^−/−^ cells after 5-FU incubation but was almost as strong after treatment with both merosesquiterpenes (Figure 6D). Both SP and IQ induced the expression of the proapoptotic genes *BAX*, *BIM*, *FASL,* and *FASR*, with higher levels in p53-proficient HCT116 cells (Figure 6E). Other proapoptotic genes such as *BID*, *NOXA*, and *PUMA* were hardly influenced by SP and IQ (Figure A5B). Antiapoptotic genes including *cIAP1*, *BCL2*, and *BCL-XL* were only slightly regulated, which occurred to a similar extent in both isogenic HCT116 cell lines (Figure A5C,D). Only *cIAP2* was found to be upregulated in p53-proficient HCT116 cells, which was reduced in p53 k.o. cells. The expression of *survivin* was repressed by all test compounds in a p53-independent manner (Figure A5C). In summary, our findings showed that merosesquiterpenes induce apoptosis in a p53-dependent manner in HCT116 cells, which involved mainly the intrinsic mitochondrial pathway via the Bim–Bax–caspase-9–caspase-3 axis. Intriguingly, SP and IQ caused similar cell death rates independent of the p53 status despite reduced apoptosis induction in the absence of p53 (see Figure 6A).

### 3.4. Influence of Mutant p53 on Cell Death Induction

**Figure 7:**
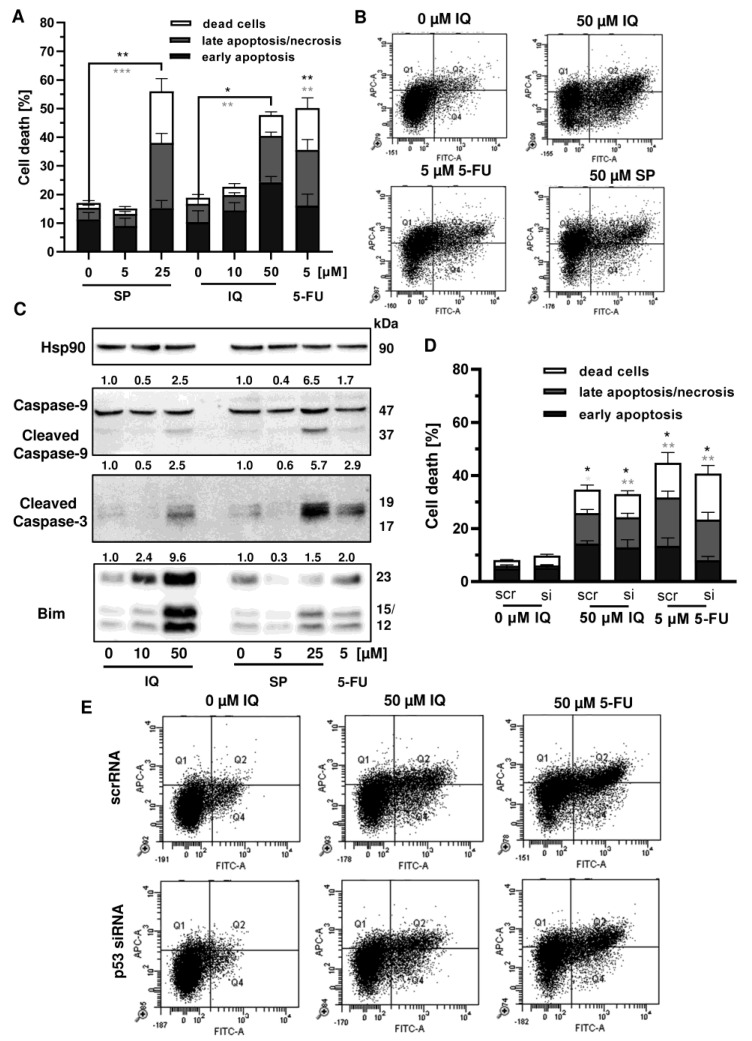
Cell death induction by merosesquiterpenes in p53-mutant CRC cells: (**A**) HT29 cells were incubated with increasing concentrations of SP or IQ, and 5-FU was included as positive control. Cell death induction was assessed by Annexin V-FITC/PI staining and flow cytometry. Data are depicted as mean + SEM (*n* = 5). Not significant (ns): *p* > 0.05; * *p* < 0.05; ** *p* < 0.01; *** *p* < 0.001; (**B**) representative dot plots of (**A**); (**C**) HT29 cells were exposed to increasing concentrations of IQ or SP for 48 h, and 5-FU was included as positive control. Samples were separated by SDS–PAGE, followed by Western blot analysis of cleaved caspase-3, cleaved caspase-9, and Bim. Hsp90 served as loading control. Representative blots are shown including the densitometric evaluation; (**D**) knockdown of p53 in HT29 cells. The cells were transfected with p53 siRNA or scrambled RNA, followed by treatment with 50 µM IQ and 5 µM 5-FU. Cell death induction was analyzed as described in panel A. Data are given as mean + SEM (*n* = 3). Not significant (ns): *p* > 0.05; * *p* < 0.05; ** *p* < 0.01; (**E**) representative dot plots of (**D**).

Given that p53 is frequently mutated in CRC, we wanted to know whether mutant p53 affects the sensitivity of CRC cells towards merosesquiterpene treatment. First, Annexin V/PI-staining was performed in p53-mutated HT29 cells and showed induction of apoptotic and necrotic cell death by SP and IQ after 48 h (Figure 7A,B). Western blot analysis revealed caspase-9 and caspase-3 cleavage following treatment with SP and IQ, which was also observed upon exposure to 5-FU (Figure 7C). Furthermore, both merosesquiterpenes caused induction of the proapoptotic protein Bim (Figure 7C). In order to characterize the role of mutant p53, a siRNA-mediated p53 knockdown was carried out in HT29 cells. As shown before, IQ caused substantial levels of cell death similar to the 5-FU treatment (Figure 7D,E). In contrast to HCT116 cells, downregulation of p53 had no impact on cell death induction triggered by IQ in HT29 cells (Figure 7D,E). Moreover, 5-FU-dependent cell death only slightly decreased upon p53 downregulation, which also contrasts with the results obtained in isogenic HCT116 cells (see Figure A4). Taken together, these findings provide evidence that merosesquiterpenes eliminate CRC cells independent of mutant p53 expression via apoptosis.

### 3.5. Therapeutic Efficacy of Merosesquiterpenquinone in Murine Tumor Organoids

**Figure 8:**
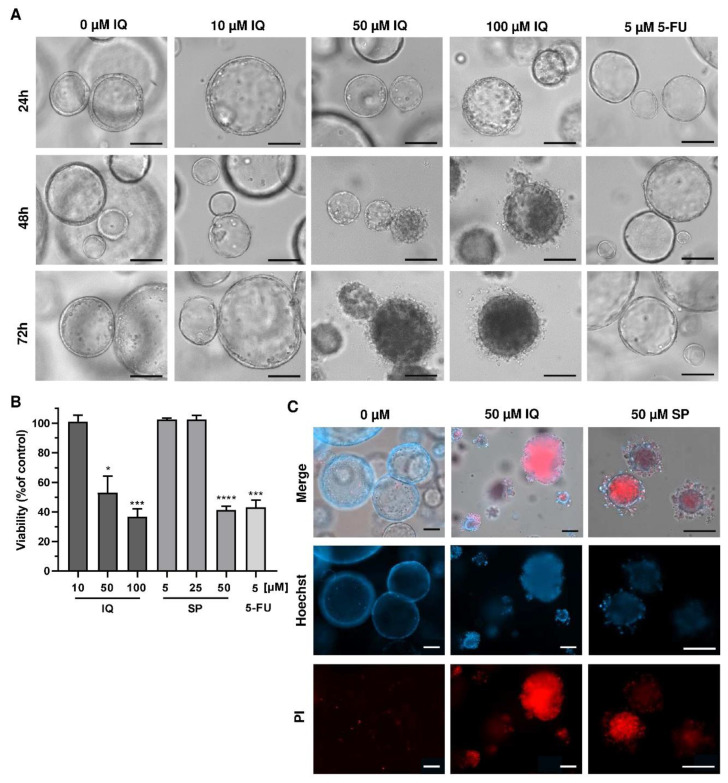
Effects of merosesquiterpenes on intestinal tumor organoids: (**A**) tumor organoids were treated with increasing concentrations of IQ, SP, 5-FU, or the solvent control and incubated for up to 72 h. Representative images of organoid morphology are shown. Scale bar: 100 µm; (**B**) organoid viability upon treatment as described in A determined by MTT assay. Data are given as mean + SEM (*n* ≥ 3). * *p* < 0.05; *** *p* < 0.001; **** *p* < 0.0001. (**C**) cell death staining in tumor organoids exposed to the indicated compounds for 72 h. Representative immunofluorescence pictures are depicted. Scale bar: 100 µm.

Finally, we translated our findings to a murine tumor organoid model. Tumor organoids were exposed to increasing concentrations of SP and IQ for up to 72 h, and 5-FU was included as the positive control. While low concentrations of the merosesquiterpenes had no impact on tumor organoid morphology and viability, higher concentrations caused substantial morphological alterations already after 24 h, culminating in complete disruption of tumor organoids after 72 h (Figure 8A, top vs. bottom panel).

**Figure 9:**
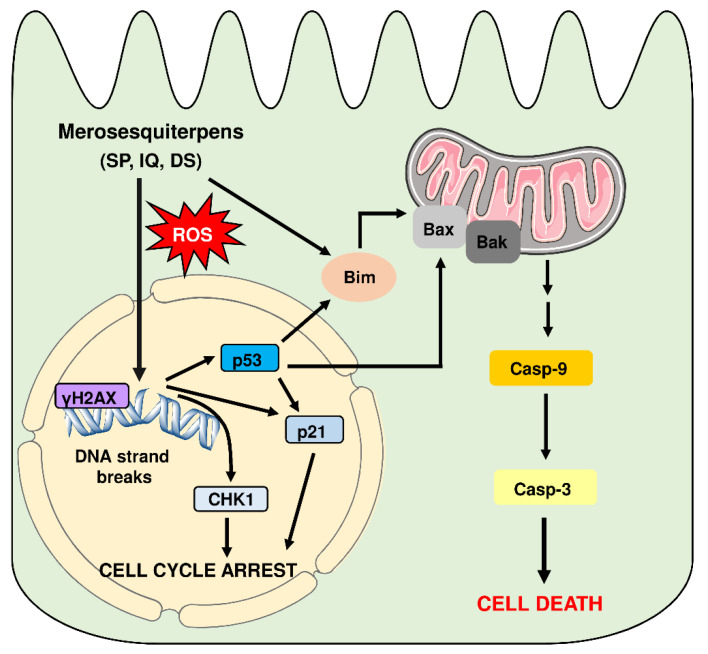
Model of cell death induction triggered by genotoxic merosesquiterpens and involved key players. Merosesquiterpenes cause DNA strand break formation in CRC cells with a concomitant ROS production. This results in the activation of the DNA damage response, as attested by γH2AX formation, CHK1 phosphorylation, and accumulation of p53 and p21. Notably, p21 induction is also observed in p53-mutant CRC cells after exposure to merosesquiterpenes. Activation of p21 and CHK1 causes initial cell cycle arrest. In CRC cells with expression of WT p53, the tumor suppressor protein activates a cell death program by upregulation of Bax, FasR, and Bim as the main proapoptotic factors, as demonstrated in isogenic p53 knockout cells. This finally results in mitochondrial apoptosis via caspase-9 and caspase-3 in p53 WT CRC cells after treatment with merosesquiterpenes. Interestingly, induction of the cell cycle regulator p21 occurs independently of the p53 status, as attested in CRC cells with mutant p53 and p53 knockout. Another important finding is that Bim induction and mitochondrial cell death are also executed in p53-mutated CRC cells after treatment with merosesquiterpens. This figure was created using Servier Medical Art templates, which are licensed under a Creative Commons Attribution 3.0 Unported License; https://smart.servier.com, accesse on 18 May 2021.

This correlated very well with the concentration-dependent decrease in tumor organoid viability as determined by the MTT assay (Figure 8B). In support of these findings, tumor organoids were highly positive for the cell death marker PI after treatment with IQ and SP, which was visualized by fluorescence microscopy (Figure 8C). In summary, these experiments showed that merosesquiterpenes not only caused cell death in established CRC cell lines but also efficiently kill tumor organoids. The major findings of this study have been summarized in a scheme shown in Figure 9.

## 4. Discussion

In this study, the therapeutic potential of numerous merosesquiterpenes was evaluated in CRC cell lines and tumor organoids. The most promising compounds were SP, IQ, and DS, with potent cytotoxic activities in the µM range. The cytotoxicity was influenced by the chemical structure, particularly the side chains. With regard to the 20-Amino and *N*-alkyl rearranged drimane sesquiterpenes, an unsubstituted amino group (i.e., SP) was responsible for the high cytotoxic activity, while an amino group substituted with an alkyl- or aromatic side chain reduced cytotoxicity. This also holds true for the sesquiterpenes with a 20-Methoxy rearranged drimane skeleton, i.e., IQ was more potent than its oxohexyl-substitued derivative quintaquinone. Interestingly, the compound 3-farnesyl- 2-hydroxy-5-methoxyquinone with an open chain configuration was completely inactive, suggesting a toxicity mechanism beyond ROS generation by the (hydro)quinone structure (see paragraph below). Another factor potentially modulating the cytotoxicity of merosequiterpenes is the enzyme NQO1 (NAD(P)H:quinone oxidoreductase 1), which catalyzes the conversion of quinones to hydroquinones [44]. This is usually considered a detoxification step but could also give rise to reactive hydroquinones. These can undergo redox cycling with the subsequent formation of electrophilic species and ROS. Thus, the expression levels of NQO1 found in cancer cells and tissues should affect their sensitivity towards merosesquiterpenes. In support of this view, inhibition of NQO1 in PC3 prostate cancer cells reduced the cytotoxic effects of IQ [45].

The growth arrest and cell death induction by merosesquiterpenes were preceded by the formation of DNA strand breaks with potent activation of the DDR, as attested by alkaline Comet assays, γH2AX formation, CHK1 phosphorylation, and p53 accumulation. Together, these genotoxicity markers demonstrate that merosesquiterpenes cause DNA damage. One plausible mechanism could involve the generation of ROS by merosesquiterpenes due to redox cycling mediated by their hydroquinone structure. In line with this notion, IQ has recently been reported to promote mitochondrial ROS production in A549 lung cancer cells [5] and to increase ROS levels in HCT116 CRC cells [4]. We were able to confirm ROS generation after treatment of HCT116 cells with high concentrations of IQ and SP, which was most prominent after 24 h. However, no effects on ROS levels were observed at lower concentrations despite clear genotoxicity, as evidenced by induction of DNA strand breaks, γH2AX formation, and DDR activation. This suggests that ROS formation is likely not the main trigger of DNA damage detected after treatment with merosesquiterpenes. Furthermore, it is conceivable that the compounds might interfere with enzymes regulating DNA topology and/or directly interact with DNA, thereby causing DNA damage. In line with the latter, an in vitro study using plasmid DNA indicated DNA cleavage by the hydroquinone species [45]. This is also in agreement with the finding that an open-chain sesquiterpene quinone displays no cytotoxicity despite being capable of redox cycling.

The merosesquiterpenes caused cell cycle arrest in G1 and/or G2/M phase, depending on the concentrations and incubation periods in CRC cells. Consistent with this finding, phosphorylation of CHK1 and upregulation of p21 were detected. CHK1 is involved in the regulation of cell cycle checkpoints in S, G2, and M-phase [46], while p21, as a CDK inhibitor, halts G1/S transition and G2/M transition [47]. Interestingly, p21 induction by merosesquiterpenes also occurred in the absence of p53 and in p53-mutated cells, strongly suggesting a p53-independent regulation of p21. Indeed, several p53-independent pathways controlling p21 gene expression were described [47], including the recently identified zinc finger protein ZNF84 upon genotoxic stress [48]. Treatment of CRC cells with higher concentrations of the merosesquiterpenes caused a significant increase in subG1 levels, indicative of apoptotic cell death, which was seen already after 24 h. Further cell death measurements, detection of caspase cleavage, and analysis of proapoptotic and antiapoptotic gene expression clearly demonstrated apoptosis induction in the CRC cell models. Our findings (caspase-9 and caspase-3 cleavage, upregulation of *BAX* and *BIM*) indicate mitochondrial apoptosis as a major cell death pathway. This was clearly dependent on p53 in CRC cells with the expression of WT p53 but also occurred in CRC cells with a hot spot mutation in p53. This is an important aspect since p53 is inactivated in the vast majority of human CRC cell lines and in more than 40% of human sporadic CRC cases [49,50]. Interestingly, the observed upregulation of the proapoptotic BH3-only protein Bim occurred not only in CRC cells with p53 WT expression but to a similar extent also in p53-mutated CRC cells, as shown by Western blot analysis. Bim can stimulate apoptosis by directly activating Bax and Bak, as well as by antagonizing antiapoptotic Bcl-2 proteins [51]. It is further worth mentioning that the selected merosesquiterpenes kill CRC cells and also display comparable cytotoxicity in tumor organoids, as shown herein. Aberrant activation of the WNT signaling pathway is a hallmark of sporadic and hereditary CRC [52]. This is primarily caused by mutations of the APC tumor suppressor gene, which regulates the degradation of the key effector molecule β-catenin [52]. Interestingly, IQ was shown to suppress the WNT/β-catenin signaling pathway in multiple myeloma cells via downregulation of β-catenin levels [53]. Thus, the deregulated WNT signaling in CRC cells might represent an additional target of merosesquiterpenes.

Another contributor to cell death induction by merosesquiterpenes might be endoplasmic reticulum (ER) stress. It was reported that IQ causes the upregulation of C/EBP homologous protein (CHOP), also denoted as DNA damage-inducible gene 153 (GADD153), in prostate cancer cells [2]. CHOP is known to be activated by ER stress and DNA damage [54]. Furthermore, CHOP can induce mitochondrial apoptosis via upregulation of the BH3-only proteins Puma and Bim [54], the latter of which was also found in our experiments. Interestingly, another study indicated IQ-dependent upregulation of CHOP in HCT116 cells via ERK and MAPK signaling, which depended on ROS formation [4]. These effects could therefore contribute to the mitochondrial apoptosis observed in our CRC cell models following exposure to the different merosesquiterpenes.

To date, little information is available on the pharmacokinetics and the potential toxicity profile of merosesquiterpenes in vivo. With regard to pharmacokinetics, a recent study performed in rats reported an oral bioavailability of 45% after administration of 10 mg IQ /kg body weight (b.w.) [55]. The maximum plasma concentration determined for single oral administration was 0.94 µg/mL, which corresponds to approximately three µM. Further experiments with i.v. injection of only 2 mg IQ/kg b.w. revealed an area under the curve (AUC) of 1.46 µg × h/mL, as compared to an AUC of 3.39 µg × h/mL, after oral administration of 10 mg/kg b.w. This suggests that i.v. injection of 10–20 mg IQ/kg b.w. yields plasma concentrations of approximately 6–12 µM. Given that SP and DS display similar pharmacokinetics, plasma concentrations with cytotoxic activity in CRC cells could thus be reached in vivo. Nevertheless, basic animal studies incorporating dose–response curves and comparing different routes of administration are clearly required. This holds also true for preclinical toxicity studies to obtain detailed information on the safety profile of the compounds, which will ultimately determine their applicability and therapeutic range. Finally, it is tempting to speculate that the merosesquiterpenes identified in this study may synergize with established anticancer drugs used in CRC therapy, as has been previously demonstrated for the combination of the natural disulfide compound LA with 5-FU or doxorubicin [34,39].

## 5. Conclusions

Taken together, we identified three natural merosesquiterpenes with potent cytotoxic activity in different human CRC cell lines independent of their p53 status. The underlying mechanism involved DNA strand break formation and activation of the DNA damage response, followed by cell cycle arrest and mitochondrial apoptotic cell death. Importantly, these findings were corroborated in murine intestinal tumor organoids, suggesting merosesquiterpenes as promising building blocks in CRC chemotherapy.

## Figures and Tables

**Table 1:** Primers used for qPCR analysis.

qPCR Target	Forward Primer (5′-3′)	Reverse Primer (5′-3′)
ACTB	TGGCATCCACGAAACTACC	GTGTTGGCGTACAGGTCTT
BAX	CAGAAGGCACTAATCAAG	ATCAGATGTGGTCTATAATG
BCL2	TTCAGAGACAGCCAGGAGAAA	AGTACCTGAACCGGCACCT
BCL-X_L_	AAGCGTAGACAAGGAGAT	TAGGTGGTCATTCAGGTAA
BID	GTGTGGATGATATGAAGGC	GAAGACAGGCTGGAAGATA
BIM	CCAAATGGCAAAGCAACCTTCTG	CTGTCAATGCATTCTCCACACC
cIAP1	TTCCCAGGTCCCTCGTATCA	CCGGCGGGGAAAGTTGAATA
cIAP2	TCACTCCCAGACTCTTTCCA	CCCCGTGTTCTACAAGTGTC
FASL	GGGATGTTTCAGCTCTTCCA	TAAATGGGCCACTTTCCTCA
FASR	TTATCTGATGTTGACTTGAGTAA	GGCTTCATTGACACCATT
GAPDH	CATGAGAAGTATGACAACAG	ATGAGTCCTTCCACGAT
MDM2	ATCTTGATGCTGGTGTAA	AGGCTATAATCTTCTGAGTC
NOXA	TCTTCGGTCACTACACAAC	CCAACAGGAACACATTGAAT
p21	ACCATGTCAGAACCGGCTGGG	TGGGCGGATTAGGGCTTC
PUMA	TAAGGATGGAAAGTGTAG	TTCAGTTTCTCATTGTTAC
Survivin	ATGACTTGTGTGTGATGA	GTTTGTGCTATTCTGTGAA

**Table 2:** IC_50_ values of merosesquiterpenes analyzed in three CRC cell models.

Compound	HCT116	RKO	HT29
smenospongine	8 µM	15 µM	10 µM
smenospongorine	39 µM	58 µM	37 µM
smenospongiarine	-	n.d.	n.d.
smenospongidine	80 µM	n.d.	n.d.
ilimaquinone	27 µM	43 µM	13 µM
5-epi-ilimaquinone	47 µM	n.d.	n.d.
quintaquinone	80 µM	n.d.	n.d.
cyclospongiaquinone-1	79 µM	n.d.	n.d.
smenodiol	31 µM	66 µM	42 µM
dactylospontriol	19 µM	40 µM	29 µM
3-farnesyl-2-hydroxy-5-methoxyquinone	-	n.d.	n.d.

n.d. = not determined.

## Data Availability

All datasets were included in the main text and in Appendix A. Raw datasets are available from the corresponding author upon reasonable request.

## Supplementary Materials

The following are available online at https://www.mdpi.com/article/10.3390/cancers13133282/s1, Supplementary Material and Methods: isolation and characterization of merosesquiterpenes, Figure S1: ^1^H-NMR of smenospongine (400 MHz, CD_3_OD), Figure S2: ^1^H-NMR of smenospongorine (500 MHz, C_6_D_6_), Figure S3: ^1^H-NMR of smenospongiarine (500 MHz, C_6_D_6_), Figure S4: ^1^H-NMR of smenospongidine (500 MHz, C_6_D_6_), Figure S5: ^1^H-NMR of ilimaquinone (500 MHz, C_6_D_6_), Figure S6: ^1^H-NMR of 5-*epi*-ilimaquinone (500 MHz, C_6_D_6_), Figure S7: ^1^H NMR of quintaquinone (500 MHz, C_6_D_6_), Figure S8: ^1^H-NMR of cyclospongiaquinone-1 (500 MHz, C_6_D_6_), Figure S9: ^1^H-NMR of smenodiol (500 MHz, C_6_D_6_), ^1^H-NMR of dactylospontriol (500 MHz, C6D6), Figure S10: ^1^H-NMR of dactylospontriol (500 MHz, C_6_D_6_), Figure S11: ^1^H-NMR of 3-farnesyl-2-hydroxy-5-methoxyquinone (500 MHz, C_6_D_6_), Figure S12: uncropped western blot images of HCT116 cells shown in Figure 3D, Figure S13: uncropped western blot images of HT29 cells shown in Figure 3D, Figure S14: uncropped western blot images of HCT116 cells and HT29 cells displayed in Figure 6C and Figure 7C, Figure S15: uncropped western blot images of HCT116 cells displayed in Figure A5A.
