# Supplementary material for: Natural Merosesquiterpenes Activate the DNA Damage Response via DNA Strand Break Formation and Trigger Apoptotic Cell Death in p53-Wild-Type and Mutant Colorectal Cancer
Source: Cancers (Basel). 2021 Jun 30;13(13):3282. doi: 10.3390/cancers13133282 (PMC8268692; doi:10.3390/cancers13133282)

cancers

MDPI

*Supplementary Information*

# Natural Merosesquiterpenes Activate the DNA Damage Response via DNA Strand Break Formation and Trigger Apoptotic Cell Death in p53-Wild-type and Mutant Colorectal Cancer

**Apisada Jiso, Philipp Demuth, Madeleine Bachowsky, Manuel Haas, Nina Seiwert, Daniel Heylmann, Birgit Rasenberger, Markus Christmann, Lea Dietrich, Thomas Brunner, Riyanti, Till F. Schäberle, Anuchit Plubrukarn, and Jörg Fahrer**

## Supplementary Material & Methods

### Isolation and characterization of Merosesquiterpenes

Merosesquiterpenes were obtained from two sponge species, an Indonesian *Haliclona* sp. BL-3, as well as a Thai *Verongula* cf. *rigida* Esper.

Smenospongine was isolated before from sponge *Dactylospongia elegans* collected from Towo'e Beach Tahuna Bay, Sangihe Islands. For reisolation of smenospongine, the sponges *Haliclona* sp. BL-3, collected from Batulewehe's coral reef Sangihe Island, was used. Therefore, the *Haliclona* specimen was extracted with MeOH (shake at 140 rpm, 30 °C, overnight). Upon removal of the solvent to dryness, the extract was purified using an HPLC system (Shimadzu Deutschland GmbH, Duisburg, Germany) equipped with a reverse-phase column (EC Gravity C18, 250 × 10 mm) and eluted with a gradient 6:4 MeCN/$H_2O$ + 0.01% TFA to 8.5:1.5 MeCN/$H_2O$ + 0.01% TFA, over 55 min, flow rate 3.0 mL/min), yielding five fractions (M1-M5). Fraction M3 was purified by reverse-phase HPLC column (gradient 55:45 MeCN/$H_2O$ + 0.01% TFA to 8:2 MeCN/$H_2O$ + 0.01% TFA, over 75 min, flow rate 3.0 mL/min) to yield **smenospongine** (2.2 mg).

The sponge *Verongula* cf. *rigida* Esper was collected from Koh Ha Islets, Krabi Province, Thailand, in two separate expeditions—one in 2010, and the other in 2014. The specimen from the 2010 expedition (1.8 kg, wet weight) was extracted with 1:1 MeOH/EtOAc (2.5 L) and EtOAc (3 × 2.5 L). The extract (23 g) was partitioned with hexane, $CH_2Cl_2$, and *n*-BuOH, respectively. An aliquot of the hexane extract (2.6 g) was chromatographed over a Sephadex LH-20 column (MeOH) and an HPLC system (Waters 1525 binary solvent delivery system with Waters 2998 photodiode array detector, a Rheodyne 7125 injection port, and performed with Empower 3 software) using a reverse-phase column (C-18, Phenomenex®, 250 × 10 mm, 10 µm) with a 9:1 MeCN/water (2.0 mL/min) to yield **dactylospontriol** (6 mg). Another aliquot of the hexane extract (11.7 g) was separated over a Sephadex LH-20 (EtOAc), $SiO_2$ (98:2 $CH_2Cl_2$/MeOH), and a $SiO_2$ HPLC columns (VertiSep GES Silica, 250×10 mm, 10 µM; 85:15 hexane/EtOAc, 2.0 mL/min) to yield **3-farnesyl-2-hydroxy-5-methoxyquinone** (6.4 mg).

The sponge specimen from the 2014 expedition (325 g, dry weight) was exhaustively extracted with hexane (5 × 3 L), $CH_2Cl_2$ (4 × 3 L), and MeOH (4 × 3 L). An aliquot of the hexane extract (3 g) was isolated with a $SiO_2$ (gradient 100:0 – 0:100 hexane/EtOAc) and Sephadex LH-20 columns (4:1 MeOH/EtOAc), yielding six pooled fractions (H1–H6). Fraction H4 was separated over $AgNO_3$-imprenated $SiO_2$ column (4:1 hexane/EtOAc) to yield **ilimaquinone** (300 mg) and **5-epi-ilimaquinone** (81 mg). Fraction H5 was isolated with a $SiO_2$ (8.5:1.5 hexane/$CHCl_3$ + 1 % v/v acetic acid), reverse-phase C-18 solid phase extraction ($CH_3CN$), and reverse-phase HPLC columns (C-18, Mightsill RP-18 GPII, 250 × 4.6 mm, 5 µm; 8.5:1.5 MeCN/water, 1.0 mL/min) to yield **quintaquinone** (1.4 mg). Another aliquot of the hexane extract (2 g) was chromatographed over a $SiO_2$ (gradient 100:0–0:100

hexane/EtOAc) and Sephadex LH-20 columns (4:1 MeOH/EtOAc) to yield six fractions (HH1–HH6). Fraction HH4 was chromatographed over reverse-phase C-18 solid phase extraction ($CH_3CN$) and separated with a reverse-phase HPLC column (8.5:1.5 $CH_3CN$/water, 1.0 mL/min) to yield **smenospongorine** (1.5 mg), **smenospongiarine** (1.2 mg), and **smenospongidine** (0.8 mg). Fraction HH5 was isolated using a reverse-phase column (7:3 $CH_3CN$/water, 1.0 mL/min) to yield **cyclospongiaquinone-1** (4.4 mg) and **smenodiol** (9 mg).

**Smenospongine**. $^1$H-NMR ($CD_3OD$, 400 MHz) *δ* 5.51 (1H, s, H-19), 4.43 (2H, brs, H2-11), 2.47 (1H, d, *J* = 13.7 Hz, H-15a), 2.41 (1H, d, *J* = 13.7 Hz, H-15b), 2.34 (1H, ddd, *J* = 13.7, 4.3, 2.7 Hz, H-3ax), 2.15 (1H, brd, *J* = 12.7 Hz, H-1eq), 2.05 (1H, brd, *J* = 13.7, 4.5 Hz, H-3eq), 1.95 (1H, m, H-2eq), 1.52 (1H, m, H-6eq), 1.43 (1H, m, H-1ax), 1.40 (1H, m, H-2ax), 1.39 (1H, m, H-8), 1.34 (3H, m, H-6ax, H2-7), 1.06 (3H, s, H3-12), 1.01 (1H, brd, *J* = 11.5 Hz, H-10), 0.98 (3H, d, *J* = 6.4 Hz, H3-13), 0.84 (3H, s, H3-14); HRESI-LCMS *m*/*z* 344.2218 $[M+H]^+$ (calcd for $C_{21}H_{29}NO_3$ 344.2220).

**Smenospongorine.** $^1$H-NMR ($C_6D_6$, 500 MHz) *δ* 6.02 (1H, brs, -NH), 5.17 (1H, s, H-19), 4.60 (1H, dd, *J* = 1.6, 1.6 Hz, H-11a), 4.56 (1H, dd, *J* = 1.6, 1.6 Hz, H-11b), 2.66 (1H, d, *J* = 13.8 Hz, H-15a), 2.57 (1H, d, *J* = 13.8 Hz, H-15b), 2.38 (1H, ddd, *J* = 12.4, 5.3, 2.2 Hz, H-1eq), 2.35 (1H, ddd, *J* = 13.7, 13.7, 5.3 Hz, H-3ax), 2.17 (1H, ddd, *J* = 13.7, 2.4, 2.4 Hz, H-3eq), 2.03 (2H, H2-22), 1.97 (1H, ddd, *J* = 11.5, 5.3, 2.4 Hz, H-2eq), 1.55 (1H, ddd, *J* = 12.4, 8.9 ,2.9 Hz, H-6eq), 1.45 (1H, ddd, *J* = 12.4, 9.7, 2.4 Hz, H-1ax), 1.44 (1H, ddd, *J* = 11.5, 5.3, 2.4 Hz, H-2ax), 1.43 (1H, ddq, *J* = 12.4, 10.0, 5.8 Hz, H-8), 1.40 (3H, overlapped, H-6ax, H2-7), 1.20 (3H, d, *J* = 5.8 Hz, H3-13), 1.12 (1H, ddd, *J* = 13.5, 6.8, 6.8 Hz, H-23), 1.06 (3H, s, H3-12), 1.04 (1H, dd, *J* = 9.7, 2.2 Hz, H-10), 0.89 (3H, s, H3-14), 0.45 (6H, d, *J* = 6.0 Hz, H3-24, H3-25); HRESIMS *m*/*z* 398.2702 $[M-H]^-$ (calcd for $C_{25}H_{36}NO_3$, 398.2686).

**Smenospongiarine.** $^1$H-NMR ($C_6D_6$, 500 MHz) *δ* 5.87 (1H, brs, -NH), 5.17 (1H, s, H-19), 4.60 (1H, dd, *J* = 1.6, 1.6 Hz, H-11a), 4.56 (1H, d, *J* = 1.6 Hz, H-11b), 2.68 (1H, d, *J* = 13.8 Hz, H-15a), 2.58 (1H, d, *J* = 13.8 Hz, H-15b), 2.38 (1H, overlapped, H-3ax), 2.37 (1H, overlapped, H-1eq), 2.19 (2H, H2-22), 2.16 (1H, dd, *J* = 13.7, 5.9 Hz, H-3eq), 1.95 (1H, ddd, *J* = 12.3, 6.0, 3.8 Hz, H-2eq), 1.53 (1H, dd, *J* = 13.5, 4.5 Hz, H-6eq), 1.44 (1H, overlapped, H-2ax), 1.43 (2H, overlapped, H-1ax, H-6ax), 1.42 (1H, dd, *J* = 5.7, 3.1 Hz, H-8), 1.40 (2H, H2-7), 1.21 (3H, d, *J* = 5.8 Hz, H3-13), 1.06 (3H, s, H3-12), 1.06 (1H, overlapped, H-24), 1.04 (1H, dd, *J* = 11.1, 1.8 Hz, H-10), 0.90 (3H, s, H3-14), 0.68 (2H, H2-23), 0.55 (6H, d, *J* = 6.6 Hz, H3-25, H3-26); HRESIMS *m*/*z* 412.2856 $[M–H]^-$ (calcd for $C_{26}H_{38}NO_3$, 412.2842).

**Smenospongidine.** $^1$H-NMR ($C_6D_6$, 500 MHz) *δ* 7.08 (2H, brd, *J* = 7.9 Hz, H-25, H-29), 7.05 (1H, brd, *J* = 7.5 Hz, H-27), 6.74 (2H, brd, *J* = 7.3 Hz, H-26, H-28), 5.91 (1H, brs, -NH), 5.12 (1H, s, H-19), 4.61 (1H, dd, *J* = 1.6, 1.6 Hz, H-11a), 4.57 (1H, brs, H-11b), 2.64 (1H, d, *J* = 13.8 Hz, H-15a), 2.53 (1H, d, *J* = 13.8 Hz, H-15b), 2.40 (2H, H2-22), 2.36 (1H, ddd, *J* =13.7, 13.7, 7.1 Hz, H-3ax), 2.33 (1H, brd, *J* = 12.6 Hz, H-1eq), 2.19 (1H, brd, *J* = 13.7 Hz, H-3eq), 2.04 (2H, H2-23), 1.94 (1H, ddd, *J* = 12.7, 5.9, 2.4 Hz, H-2eq), 1.55 (1H, dd, *J* = 13.5, 2.9 Hz, H-6eq), 1.46 (1H, overlapped, H-1ax), 1.42 (3H, overlapped, H-2ax, H2-7), 1.41 (1H, dd, *J* = 5.7, 3.1 Hz, H-8), 1.40 (1H, overlapped, H-6ax), 1.17 (3H, d, *J* = 5.4 Hz, H3-13), 1.06 (3H, s, S-14, H3-12), 1.03 (1H, dd, *J* = 11.7, 2.2 Hz, H-10), 0.88 (3H, s, H3-14); HRESIMS *m*/*z* 448.2846 $[M+H]^+$ (calcd for $C_{29}H_{38}NO_3$, 448.2842).

**Ilimaquinone.** $^1$H-NMR ($C_6D_6$, 500 MHz) *δ* 7.37 (1H, s, 17-OH), 5.08 (1H, s, H-19), 4.58 (1H, dd, *J* = 1.6, 1.6 Hz, H-11a), 4.55 (1H, dd, *J* = 1.6, 1.6 Hz, H-11b), 2.68 (1H, d, *J* = 13.7 Hz, H-15a), 2.64 (3H, s, 20-$OCH_3$), 2.55 (1H, d, *J* = 13.7 Hz, H-15b), 2.35 (1H, dddd, *J* = 13.5, 11.5, 5.5, 1.6 Hz, H-3ax), 2.32 (1H, brdd, *J* = 11.5, 2.2 Hz, H-1eq), 2.13 (1H, dddd, *J* = 13.5, 4.5, 2.2, 2.2 Hz, H-3eq), 1.93 (1H, brddd, *J* = 11.7, 5.5, 2.2 Hz, H-2eq), 1.53 (1H, ddd, *J* = 11.8, 3.1, 3.1 Hz, H-6eq), 1.44 (1H, dddd, *J* = 11.5, 11.5, 11.3, 2.2 Hz, H-1ax), 1.43 (1H, dddd, *J* = 11.7, 11.5, 2.2, 2.2 Hz, H-2ax), 1.40 (1H, ddd, *J* = 11.8, 2.4, 2.4 Hz, H-6ax), 1.38 (1H, brdd, *J* = 5.8, 1.7 Hz, H-8), 1.36 (2H, H2-7), 1.16 (3H, d, *J* = 5.8 Hz, H3-13), 1.03 (3H, s, H3-12), 0.98 (1H, dd, *J* = 11.3, 2.2 Hz, H-10), 0.86 (3H, s, H3-14); HRESIMS *m*/*z* 381.2025 $[M+Na]^+$ (calcd for $C_{22}H_{30}O_4Na$, 381.2034).

**5-*Epi*-ilimaquinone.** $^{1}$H-NMR ($C_6D_6$, 500 MHz) *δ* 7.36 (1H, s, 17-OH), 5.19 (1H, s, H-19), 4.81 (1H, dd, *J* = 1.7, 1.7 Hz, H-S-13 11a), 4.78 (1H, dd, *J* = 1.7, 1.7 Hz, H-11b), 2.72 (1H, d, *J* = 13.6 Hz, H-15a), 2.70 (3H, s, 20-$OCH_3$), 2.60 (1H, d, *J* = 13.6 Hz, H-15b), 2.43 (1H, dddd, *J* = 13.9, 13.5, 6.4, 1.7 Hz, H-3ax), 2.34 (1H, brdd, *J* = 15.4, 4.3 Hz, H-1eq), 2.12 (1H, dd, *J* = 13.9, 5.5 Hz, H-3eq), 2.05 (1H, ddd, *J* = 13.9, 3.2, 3.2 Hz, H-6eq), 1.96 (1H, dddd, *J* = 15.4, 14.6, 6.4, 6.4 Hz, H-1ax), 1.83 (1H, dddd, *J* = 13.5, 13.5, 5.5, 5.5 Hz, H-2eq), 1.64 (1H, dddd, *J* = 13.5, 13.5, 11.7, 2.3 Hz, H-2ax), 1.61 (1H, ddd, *J* = 13.4, 6.4, 3.2 Hz, H-7eq), 1.37 (1H, ddd, *J* = 6.4, 6.4, 3.5 Hz, H-8), 1.36 (1H, dd, *J* = 6.4, 4.3 Hz, H-10), 1.22 (1H, dddd, *J* = 13.4, 7.7, 3.5, 3.2 Hz, H-7ax), 1.12 (3H, d, *J* = 6.4 Hz, H3-13), 1.10 (1H, ddd, *J* = 13.9, 7.7, 3.5 Hz, H-6ax), 1.10 (3H, s, H3-12), 1.00 (3H, s, H3-14); HRESIMS *m/z* 381.2047 $[M+Na]^+$ (calcd for $C_{22}H_{30}O_4Na$, 381.2034).

**Quintaquinone.** $^{1}$H-NMR ($C_6D_6$, 500 MHz) *δ* 4.59 (1H, dd, *J* = 1.6, 1.6 Hz, H-11a), 4.55 (1H, brs, H-11b), 3.78 (3H, s, 20-$OCH_3$), 2.66 (1H, d, *J* = 13.6 Hz, H-15a), 2.51 (1H, d, *J* = 13.6 Hz, H-15b), 2.35 (1H, dddd, *J* = 11.5, 11.2, 3.2, 1.6 Hz, H-3ax), 2.30 (2H, H2-22), 2.28 (1H, ddd, *J* = 11.2, 3.5, 1.8 Hz, H-1eq), 2.14 (1H, dd, *J* = 11.5, 2.2 Hz, H-3eq), 1.93 (1H, ddd, *J* = 11.2, 3.2, 2.2 Hz, H-2eq), 1.86 (2H, t, *J* = 7.3 Hz, H2-25), 1.61 (3H, s, H3-27), 1.55 (1H, brdd, *J* = 10.9, 2.6 Hz, H-6eq), 1.45 (3H, ddd, *J* = 11.2, 11.2, 2.2 Hz, H-1ax, H2-24), 1.42 (2H, ddd, *J* = 10.9, 10.9, 2.6 Hz, H-6ax, H-7eq), 1.40 (1H, dddd, *J* = 11.2, 11.2, 3.5, 2.2 Hz, H-2ax), 1.35 (1H, overlapped, H-7ax), 1.32 (1H, dq, *J* = 12.2, 6.0 Hz, H-8), 1.26 (2H, H2-23), 1.13 (3H, d, *J* = 6.0 Hz, H3-13), 1.04 (3H, s, H3-12), 0.96 (1H, dd, *J* = 11.2, 1.8 Hz, H-10), 0.86 (3H, s, H3-14); HRESIMS *m/z* 479.2763 $[M+Na]^+$ (calcd for $C_{28}H_{40}O_5Na$, 479.2763).

**Cyclospongiaquinone-1.** $^{1}$H-NMR ($C_6D_6$, 500 MHz) *δ* 5.41 (1H, s, H-19), 2.88 (3H, s, 20-$OCH_3$), 2.45 (1H, dd, *J* = 18, 4.7 Hz, H-15a), 1.97 (1H, ddd, *J* = 12.7, 3.3, 3.3 Hz, H-7eq), 1.91 (1H, dd, *J* = 18, 12.9 Hz, H-15b), 1.57 (1H, ddd, *J* = 12.7, 12.7, 4.4 Hz, H-7ax), 1.37 (1H, ddd, *J* = 13.8, 7.1, 3.6 Hz, H-2eq), 1.32 (1H, ddd, *J* = 12.8, 7.2, 3.4 Hz, H-1eq), 1.27 (1H, ddd, *J* = 13.1, 4.3, 2.9 Hz, H-6eq), 1.25 (1H, ddd, *J* = 13.9, 5.2, 3.2 Hz, H-3eq), 1.22 (1H, ddd, *J* = 13.1, 7.1, 3.5 Hz, H-6ax), 1.07 (1H, dd, *J* = 12.9, 4.7 Hz, H-9), 0.97 (1H, ddd, *J* = 13.9, 13.9, 3.8 Hz, H-3ax), 0.90 (1H, ddd, *J* = 13.8, 13.8, 3.4 Hz, H-2ax), 0.90 (3H, s, H3-13), 0.74 (3H, s, H3-12), 0.66 (3H, s, H3-11), 0.62 (1H, dd, *J* = 12.4, 2.2 Hz, H-5), 0.54 (1H, ddd, *J* = 12.8, 3.3, 3.3 Hz, H-1ax), 0.49 (3H, s, H3-14); ESIMS *m/z* 381.2037 $[M+Na]^+$ (calcd for $C_{22}H_{30}O_4Na$, 381.2034).

**Smenodiol.** $^{1}$H-NMR ($C_6D_6$, 500 MHz) *δ* 7.96 (1H, brs, H-17), 7.41 (1H, brs, H-19), 5.77 (1H, brs, 21-OH), 5.41 (1H, brs, H-7), 5.01 (1H, brs, 20-OH), 3.51 (3H, s, 22-$OCH_3$), 2.89 (1H, dd, *J* = 15.4, 9.4 Hz, H-15a), 2.69 (1H, dd, *J* = 15.4, 2.5 Hz, H-15b), 2.56 (1H, brd, *J* = 9.4 Hz, H-9), 1.94 (1H, brd, *J* =13.0 Hz, H-6eq), 1.86 (2H, overlapped, H-1eq, H-6ax), 1.64 (3H, dd, *J* = 2.2, 1.2 Hz, H3-13), 1.47 (1H, ddd, *J* = 13.0, 3.3, 3.3 Hz, H-2eq), 1.35 (2H, ddd, *J* = 13.0, 5.6, 3.3 Hz, H-2ax, H-3eq), 1.20 (1H, dd, *J* = 11.8, 5.1 Hz, H-5), 1.12 (1H, ddd, *J* = 13.0, 10.1, 3.3 Hz, H-1ax), 1.10 (1H, ddd, *J* = 13.0, 10.1, 3.3 Hz, H-3ax), 0.90 (3H, s, H3-14), 0.86 (3H, s, H3-11), 0.83 (3H, s, H3-12); HRESIMS *m/z* 373.2369 $[M+H]^+$ (calcd for $C_{23}H_{33}O_4$, 373.2370).

**Dactylospontriol.** $^{1}$H-NMR ($C_6D_6$, 500 MHz) *δ* 11.29 (1H, s, 19-OH), 7.55 (1H, s, H-17), 5.78 (1H, s, 20-OH), 5.42 (1H, s, H-7), 3.31 (3H, s, 22-$OCH_3$), 2.82 (1H, d, *J* = 15.5 Hz, H-15a), 2.65 (1H, dd, *J* = 15.5, 9.6 Hz, H-15b), 2.51 (1H, brs, H-9), 1.94 (1H, overlapped, H-6eq), 1.87 (1H, overlapped, H-6ax), 1.85 (1H, overlapped, H-1eq), 1.66 (3H, s, H3-13), 1.48 (1H, overlapped, H-2eq), 1.35 (1H, overlapped, H-2ax), 1.33 (1H, overlapped, H-3eq), 1.21 (1H, dd, *J* = 11.7, 5.0 Hz, H-5), 1.08 (1H, overlapped, H-1ax), 1.05 (1H, overlapped, H-3ax), 0.89 (3H, s, H3-14), 0.85 (3H, s, H3-11), 0.82 (3H, s, H3-12); EIMS *m/z* (% relative intensity): 388 ([M]+, 36), 197 (100), 164 (40).

**3-Farnesyl-2-hydroxy-5-methoxyquinone.** $^{1}$H-NMR ($C_6D_6$, 500 MHz) *δ* 5.48 (1H, tq, *J* = 7.1, 1.2 Hz, H-2'), 5.22 (2H, tq, *J* = 6.9, 1.4 Hz, H-6', H-10'), 5.13 (1H, s, H-6), 3.30 (2H, d, *J* = 7.1 Hz, H2-1'), 2.72 (3H, s, 5-$OCH_3$), 2.15 (2H, overlapped, H2-5'), 2.14 (2H, overlapped, H2-9'), 2.04 (2H, overlapped, H2-4'), 2.03 (2H, overlapped, H2-8'), 1.82 (3H, brs, 3'-$CH_3$), 1.67 (3H, d, *J* = 1.10 Hz, H3-12'), 1.55 (3H, s, 7'-$CH_3$), 1.54 (3H, s, 11'-$CH_3$); HRESIMS m/z 359.2211 [M+H]+ (calcd for $C_{22}H_{31}O_4$, 359.2214).

## Supplementary Figures

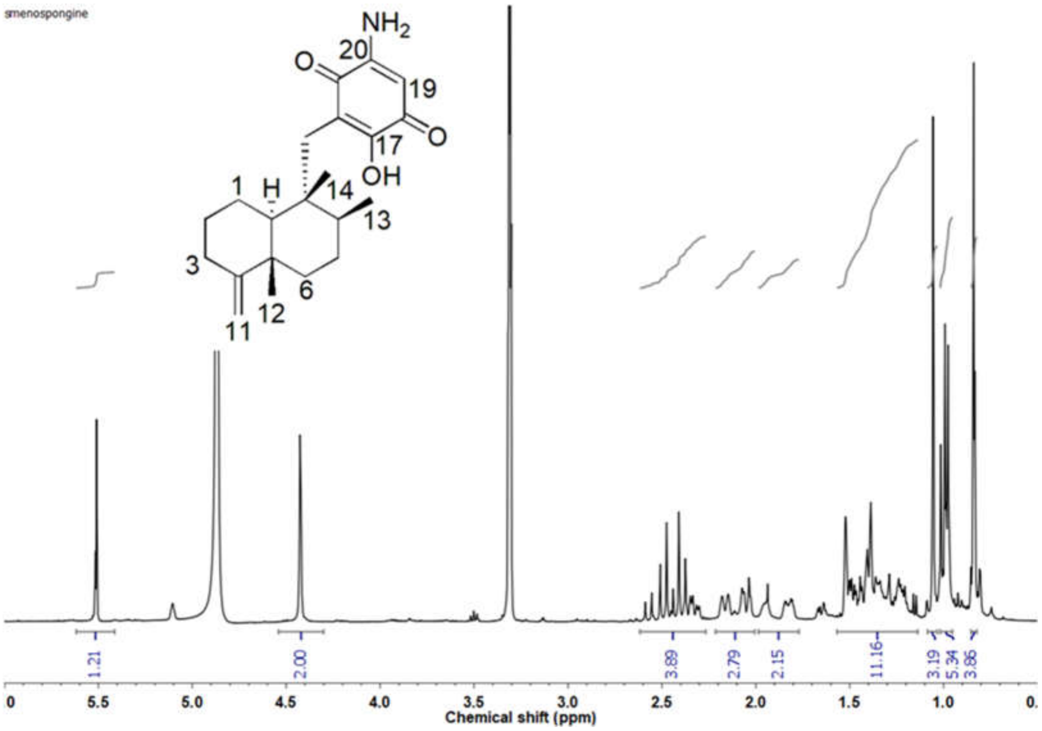


**Figure S1.** $^1$H-NMR of smenospongine (400 MHz, $CD_3OD$).

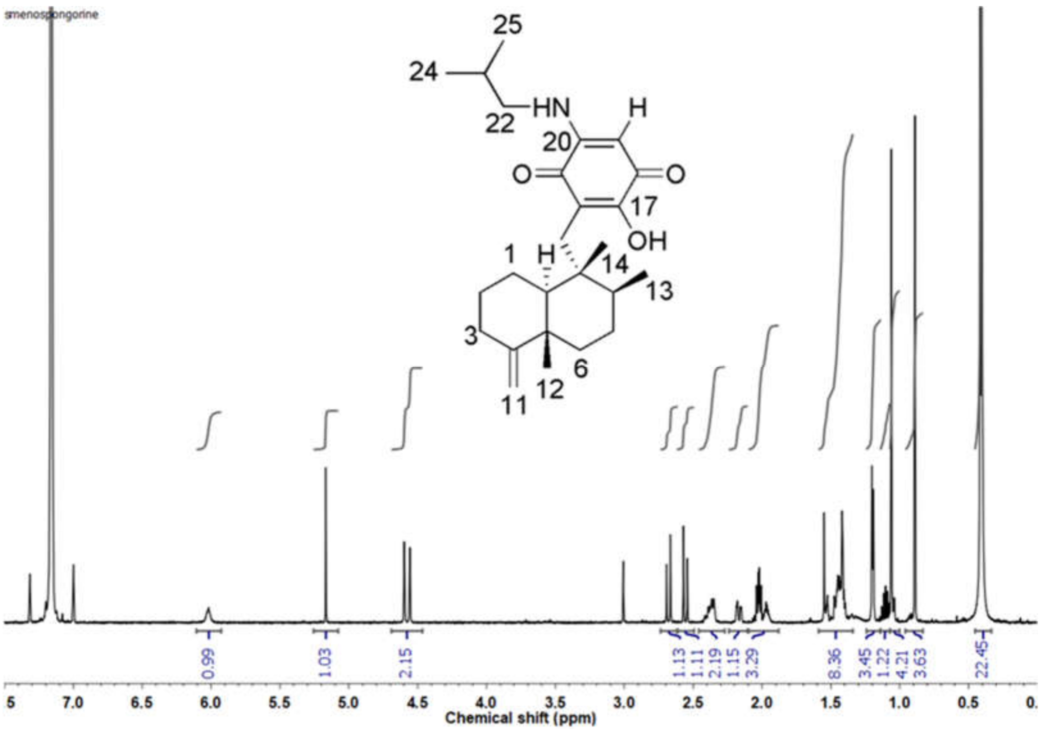


**Figure S2.** $^1$H-NMR of smenospongorine (500 MHz, $C_6D_6$).

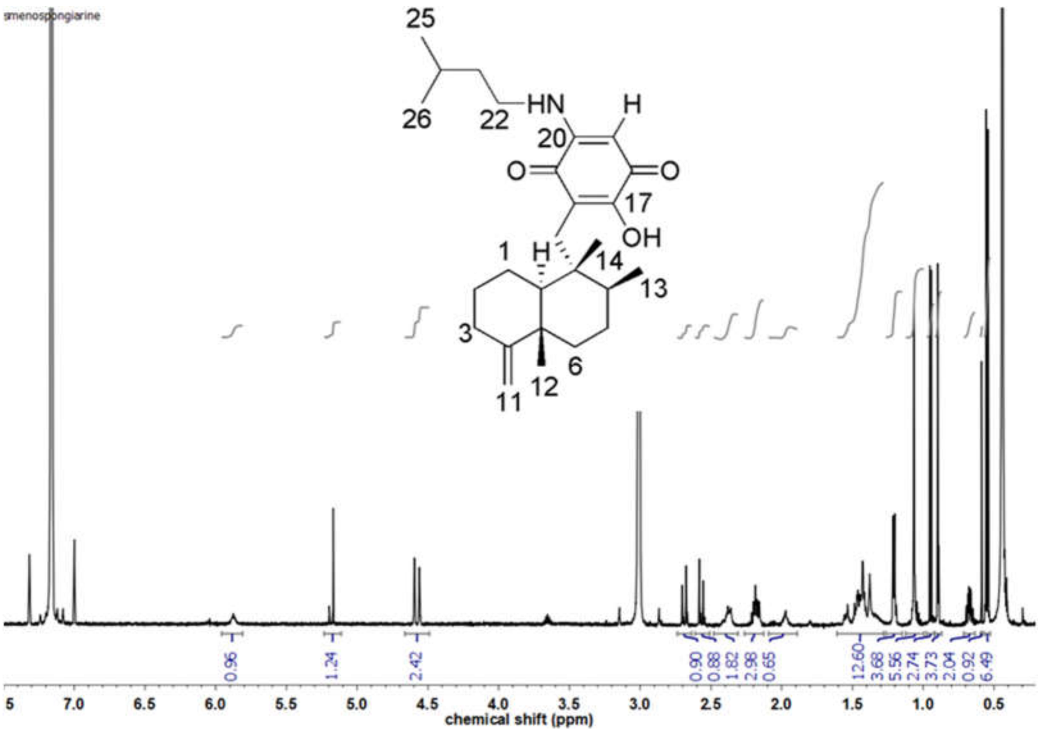

**Figure S3.** $^{1}$H-NMR of smenospongiarine (500 MHz, $C_6D_6$).

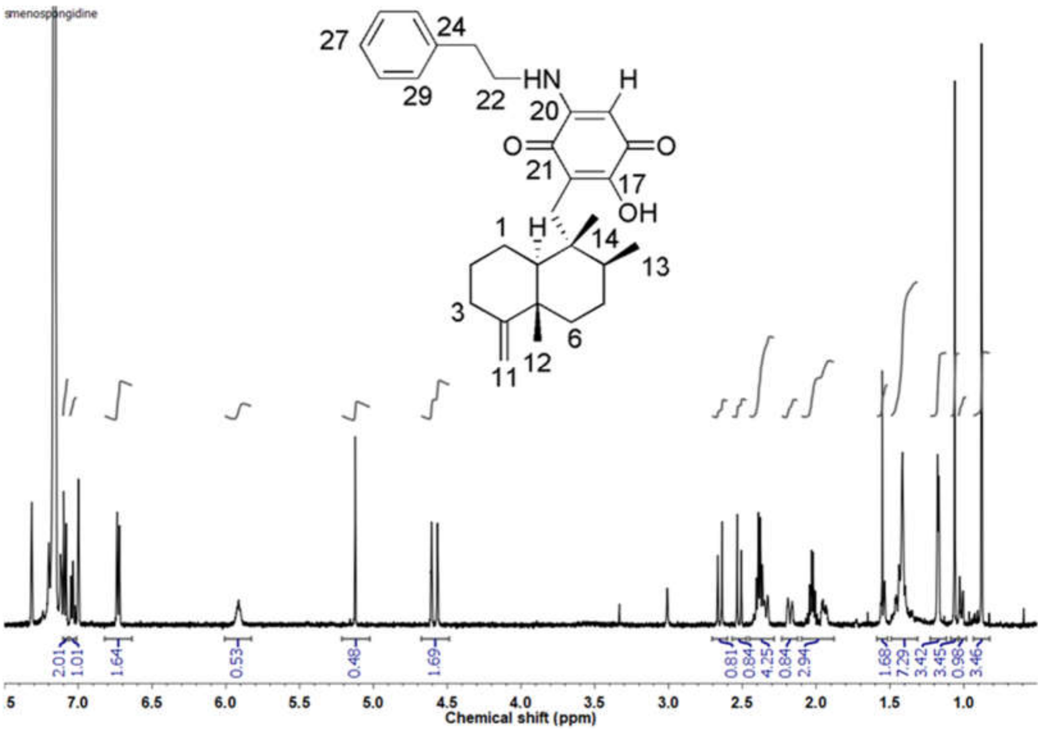

**Figure S4.** $^{1}$H-NMR of smenospongidine (500 MHz, $C_6D_6$).

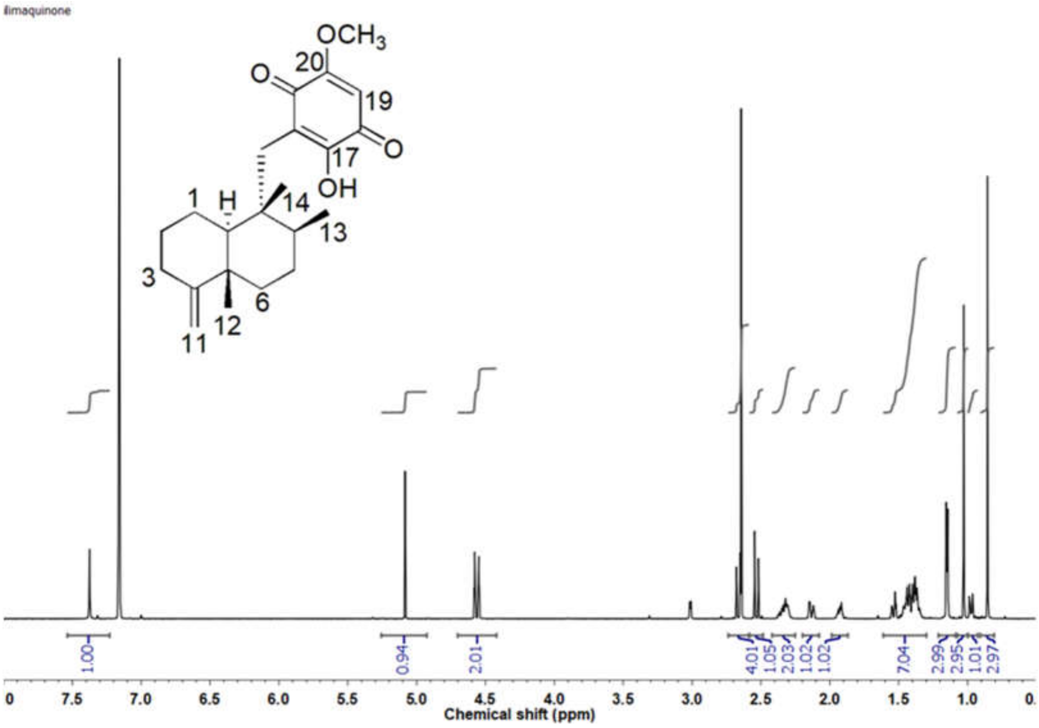


**Figure S5.** $^1$H-NMR of ilimaquinone (500 MHz, $C_6D_6$).

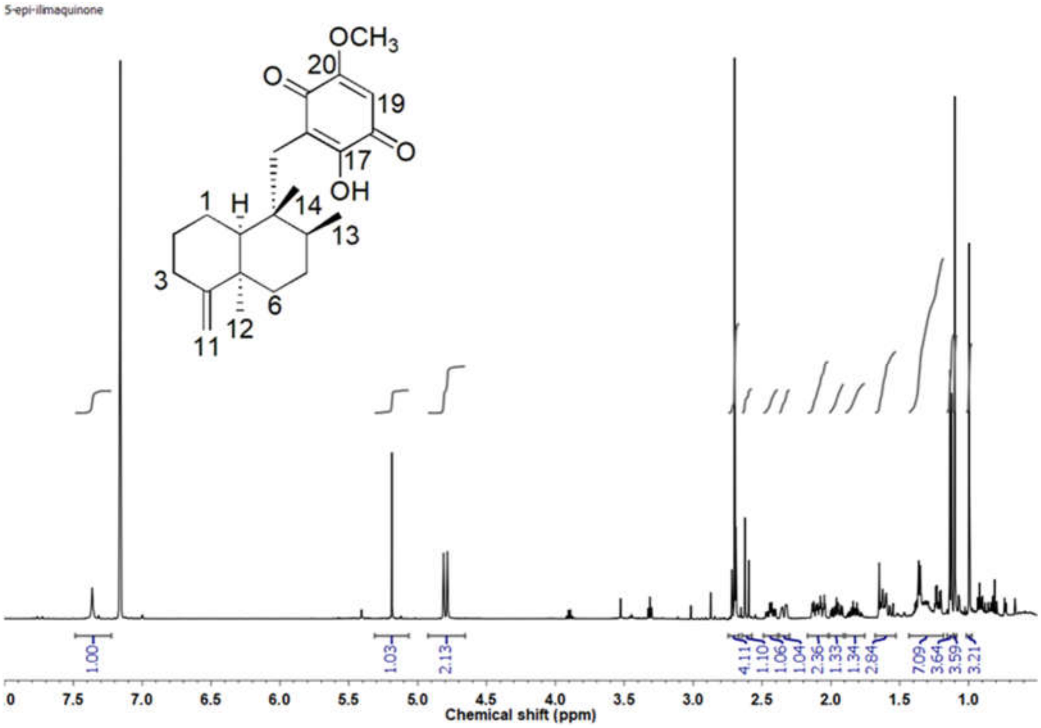


**Figure S6.** $^1$H-NMR of 5-*epi*-ilimaquinone (500 MHz, $C_6D_6$).

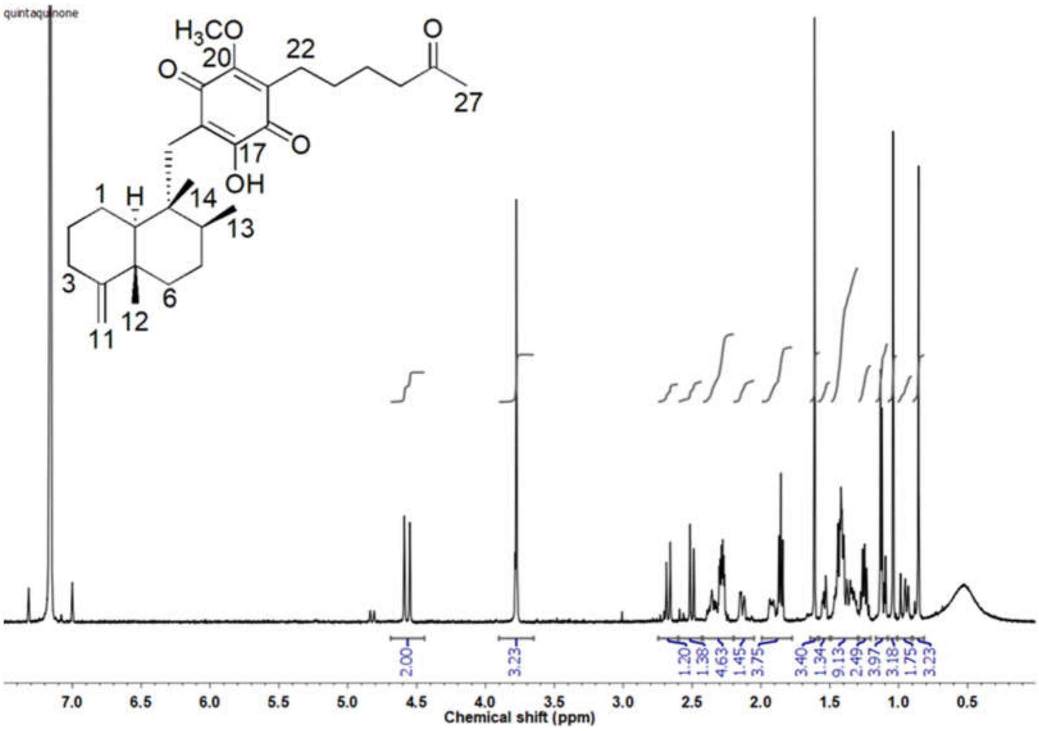


**Figure S7.** $^{1}$H-NMR of quintaquinone (500 MHz, $C_6D_6$).

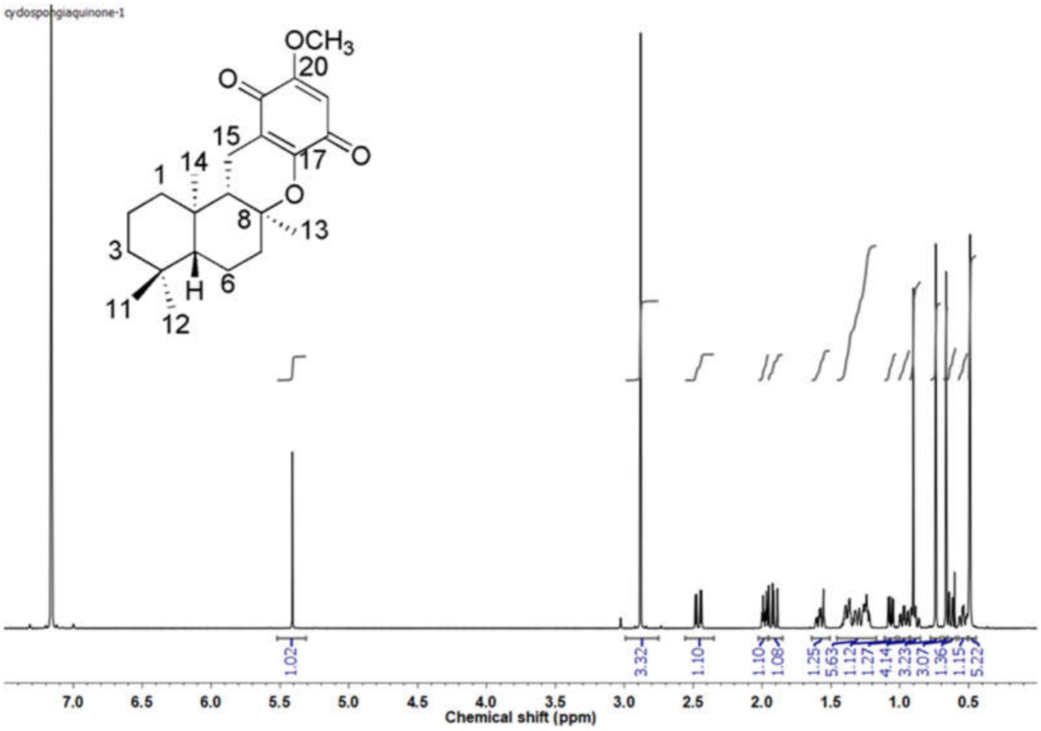


**Figure S8.** $^{1}$H-NMR of cyclospongiaquinone-1 (500 MHz, $C_6D_6$).

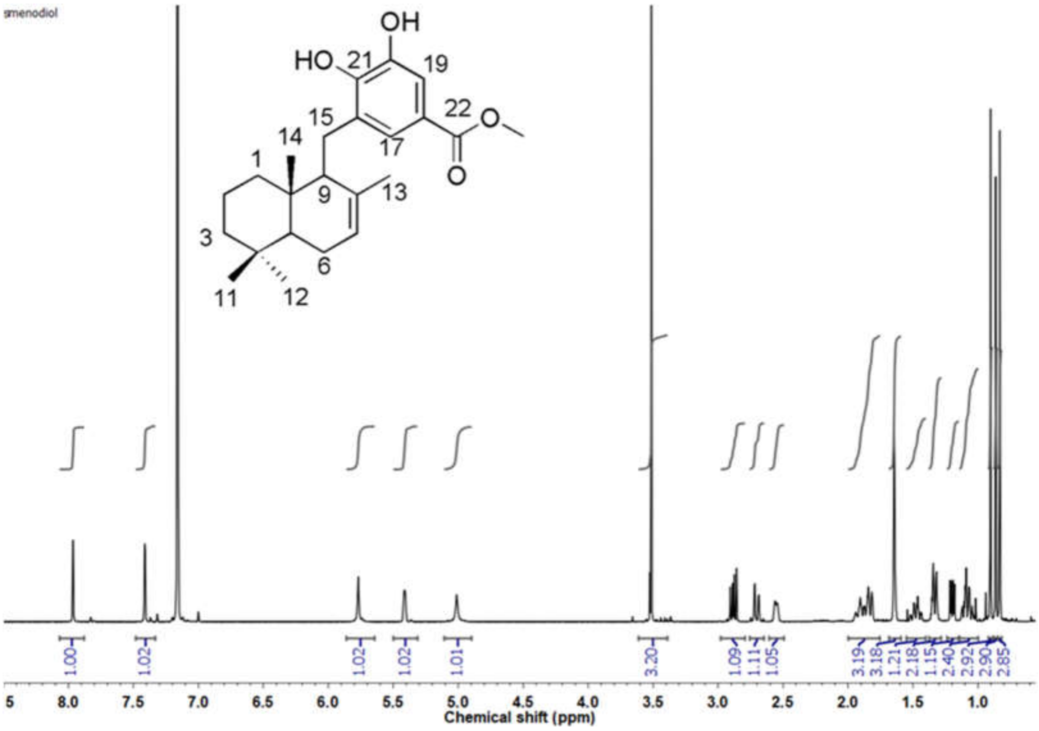


**Figure S9.** $^1$H-NMR of smenodiol (500 MHz, $C_6D_6$).

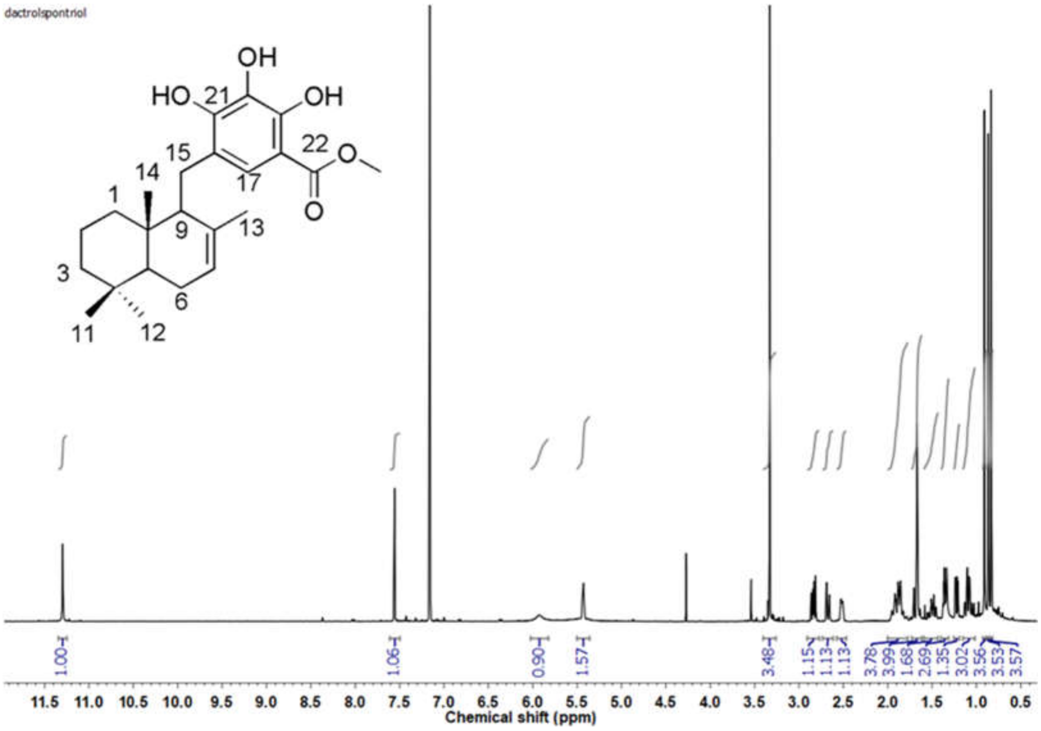


**Figure S10.** $^1$H-NMR of dactylospontriol (500 MHz, $C_6D_6$).

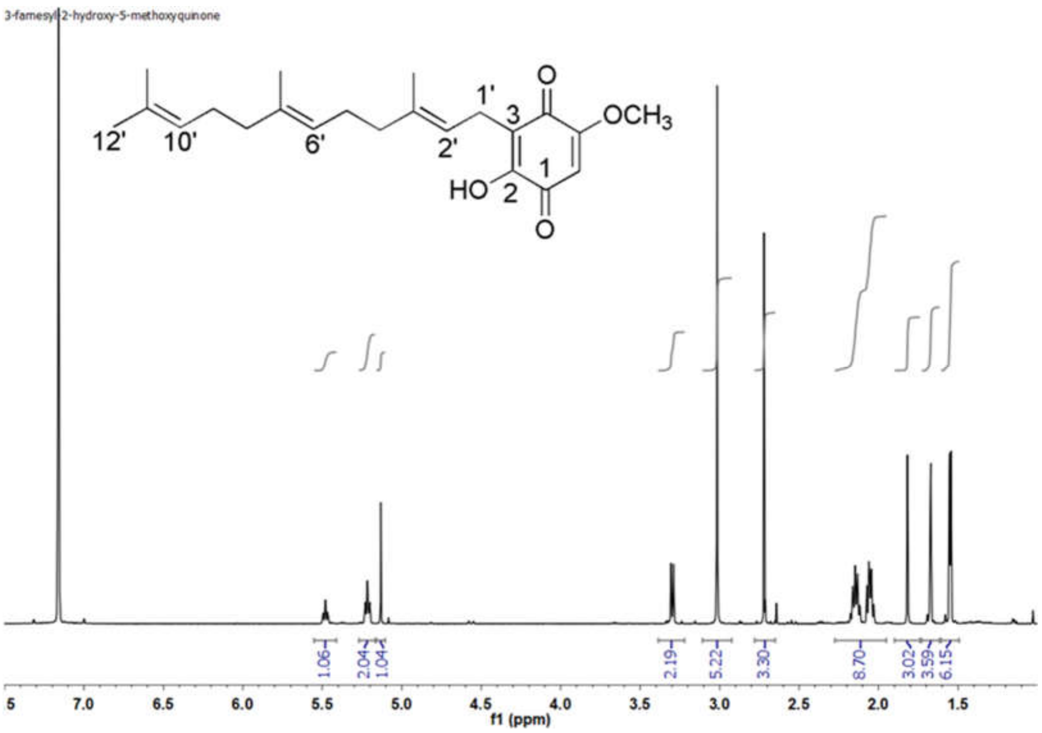


**Figure S11.** $^{1}$H-NMR of 3-farnesyl-2-hydroxy-5-methoxyquinone (500 MHz, $C_6D_6$).

## Figure S12

**Western blots HCT116 cells, Fig. 3D**

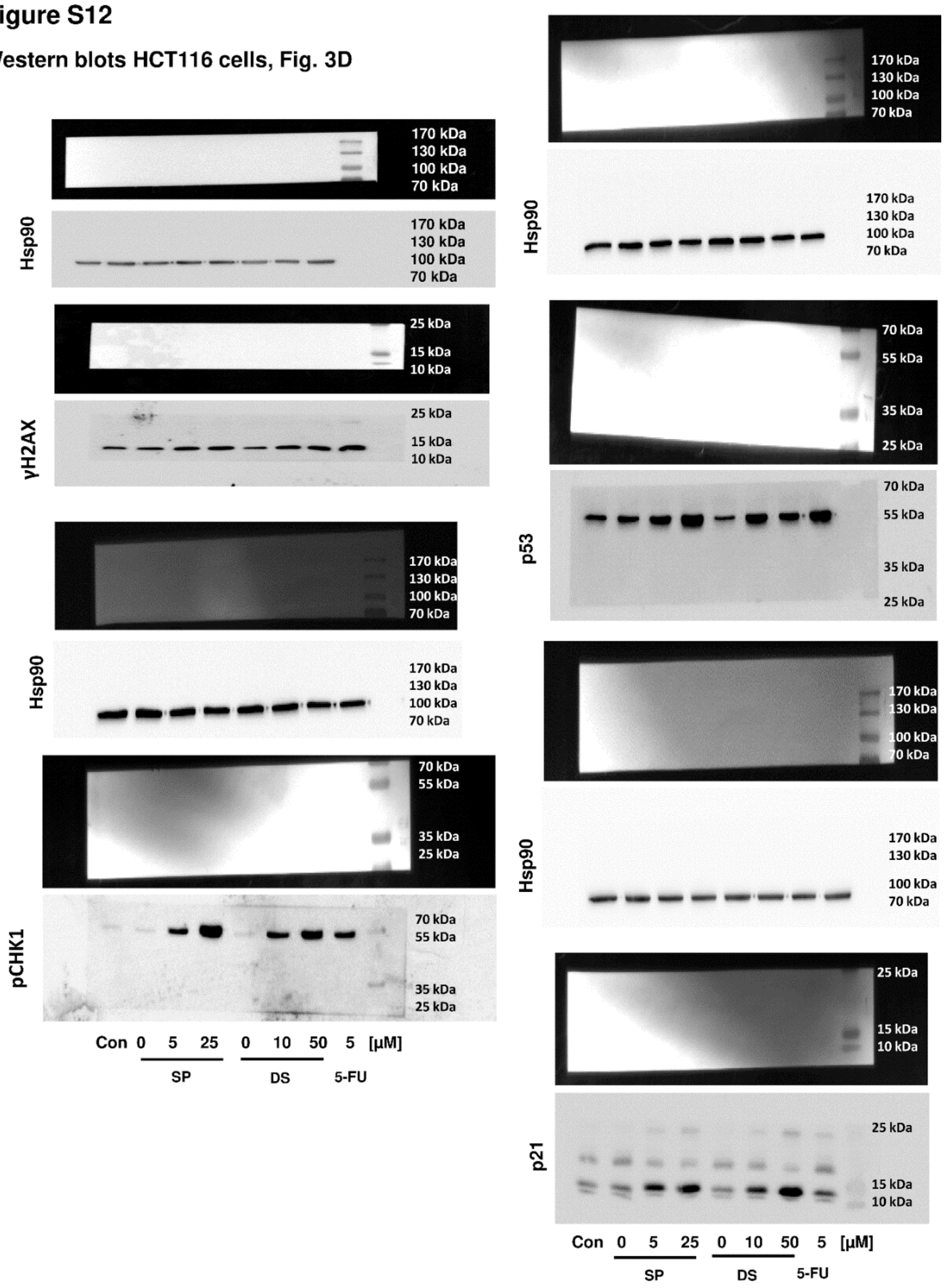

# Figure S13

## Western blots HT29 cells, Fig. 3D

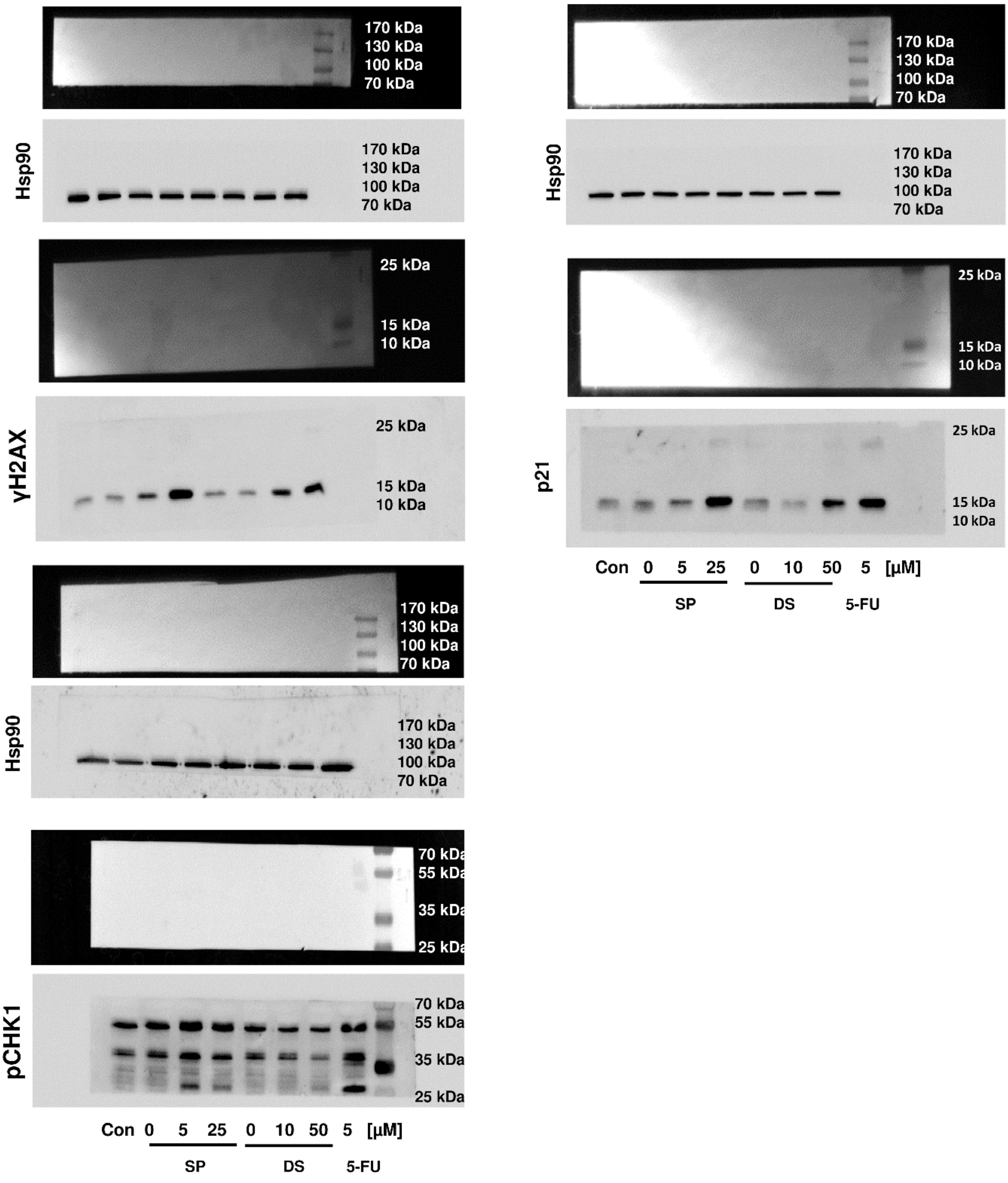

# Figure S14

## Western blots HCT116 cells, Fig. 6C and HT29 cells, Fig. 7C

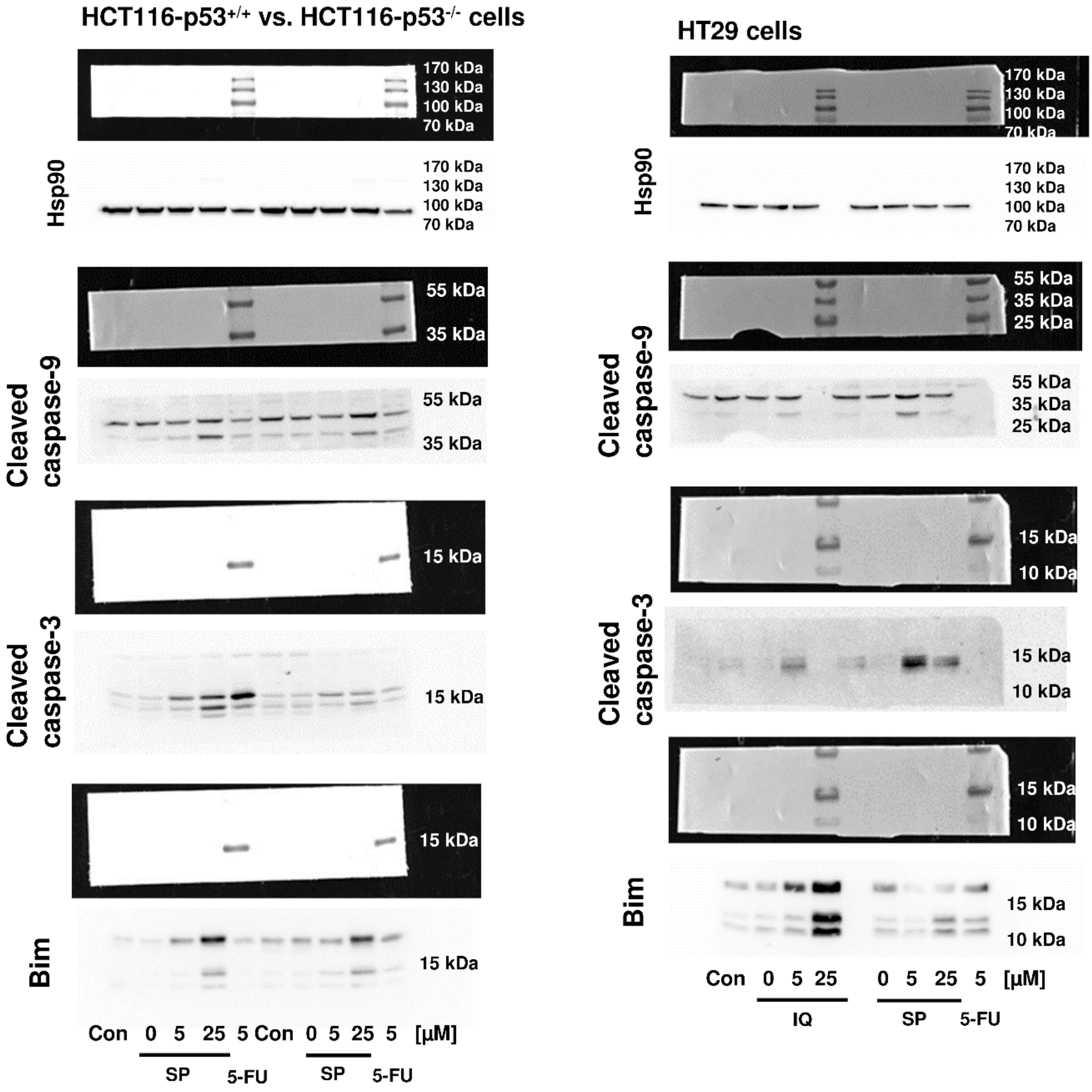

# Figure S15

## Western blots HCT116 cells, Fig. A5A

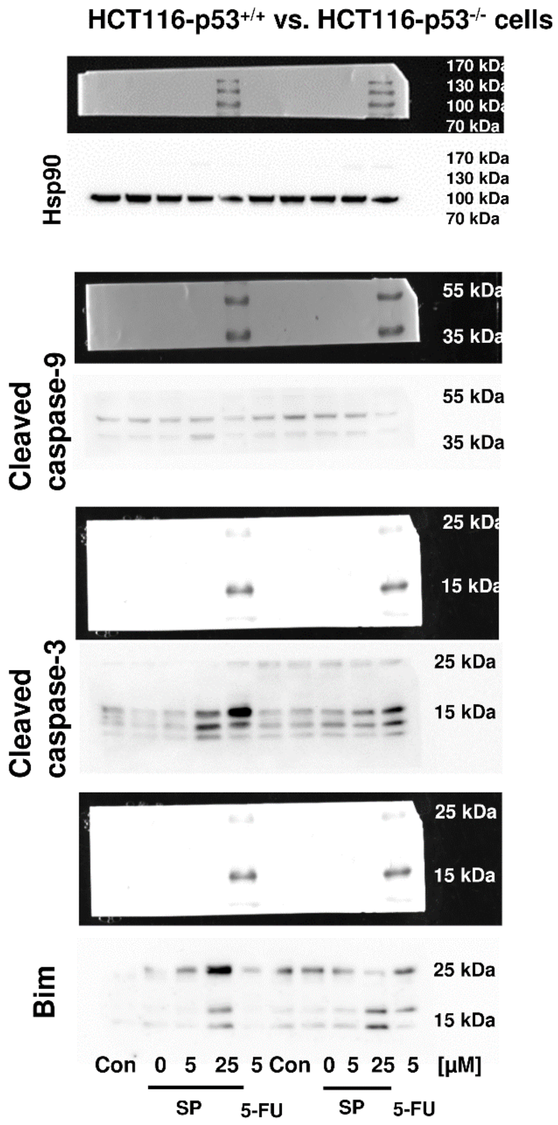

Supplement: Supplementary file 1 [file cancers-13-03282-s001.zip › cancers-1245175-supplementary.pdf]
